# Oxidative Post-translational Protein Modifications upon Ischemia/Reperfusion Injury

**DOI:** 10.3390/antiox13010106

**Published:** 2024-01-16

**Authors:** Aleksandra Binek, Celia Castans, Inmaculada Jorge, Navratan Bagwan, José Manuel Rodríguez, Rodrigo Fernández-Jiménez, Carlos Galán-Arriola, Eduardo Oliver, Mónica Gómez, Agustín Clemente-Moragón, Borja Ibanez, Emilio Camafeita, Jesús Vázquez

**Affiliations:** 1 Centro Nacional de Investigaciones Cardiovasculares (CNIC), Melchor Fernández Almagro, 3, 28029 Madrid, Spain; 2 CIBER de Enfermedades Cardiovasculares (CIBERCV), 28029 Madrid, Spain; 3 Department of Cardiology, Hospital Universitario Clínico San Carlos, Profesor Martín Lagos, s/n, 28040 Madrid, Spain; 4 Centro de Investigaciones Biológicas Margarita Salas (CIB), CSIC, Ramiro de Maeztu 9, 28040 Madrid, Spain; 5 IIS-Fundación Jiménez Díaz Hospital, Avenida Reyes Católicos, 2, 28040 Madrid, Spain

**Keywords:** myocardial infarction, ischemia/reperfusion, ischemic preconditioning, pig ischemia/reperfusion model, proteomics, post-translational modifications, oxidative post-translational modifications

## Abstract

While reperfusion, or restoration of coronary blood flow in acute myocardial infarction, is a requisite for myocardial salvage, it can paradoxically induce a specific damage known as ischemia/reperfusion (I/R) injury. Our understanding of the precise pathophysiological molecular alterations leading to I/R remains limited. In this study, we conducted a comprehensive and unbiased time-course analysis of post-translational modifications (PTMs) in the post-reperfused myocardium of two different animal models (pig and mouse) and evaluated the effect of two different cardioprotective therapies (ischemic preconditioning and neutrophil depletion). In pigs, a first wave of irreversible oxidative damage was observed at the earliest reperfusion time (20 min), impacting proteins essential for cardiac contraction. A second wave, characterized by irreversible oxidation on different residues and reversible Cys oxidation, occurred at late stages (6–12 h), affecting mitochondrial, sarcomere, and inflammation-related proteins. Ischemic preconditioning mitigated the I/R damage caused by the late oxidative wave. In the mouse model, the two-phase pattern of oxidative damage was replicated, and neutrophil depletion mitigated the late wave of I/R-related damage by preventing both Cys reversible oxidation and irreversible oxidation. Altogether, these data identify protein PTMs occurring late after reperfusion as an actionable therapeutic target to reduce the impact of I/R injury.

## 1. Introduction

Early reperfusion, the strategy of choice to halt the damage produced by heart ischemia, has dramatically reduced the mortality associated with myocardial infarction (MI); however, reperfusion paradoxically produces additional damage to the myocardium, known as ischemia/reperfusion (I/R) injury, which may account for up to 40% of infarct size [1,2]. The detrimental effects of I/R injury include myocardial damage, cardiomyocyte death, and cardiac dysfunction, resulting in cardiac arrhythmia, heart failure, and death [3]. 

Ischemia produces the accumulation of Ca^2+^, inorganic phosphate, and ADP, together with oxidative stress, which, upon reperfusion, promotes the opening of the mitochondrial permeability transition pore (MPTP), the hallmark of reperfusion-induced cardiomyocyte death [3,4]. Opening of the MPTP collapses the mitochondrial membrane potential, uncoupling oxidative phosphorylation and leading to ATP depletion and cell death [3]. MPTP opening also induces the liberation of apoptotic factors [3] and promotes a burst of additional reactive oxygen species (ROS) [5]. Endogenous alarm signals termed “danger-associated molecular patterns” [6] released from dying cardiomyocytes activate Toll-like receptor (TLR)-dependent downstream signaling pathways in vascular endothelial cells, immune cells, and fibroblasts. These pathways induce the expression of proinflammatory chemokines, cytokines, and adhesion molecules, promoting neutrophil activation and leukocyte infiltration into the infarcted area, where they phagocyte dead cells and matrix debris [7]. This inflammatory phase results in further cytokine secretion, oxidative stress, and protease release, which exacerbate myocardial and endothelial damage, thereby aggravating I/R injury. The ROS released during the re-establishment of blood flow have also been proposed to activate the NF-kB pathway, directly triggering an inflammatory response [8]. 

Recent findings challenge our current interpretation of the above-described biochemical processes at play in I/R injury. A tissue hallmark of MI is the production of edema during the early stages of ischemia [9]. Edema is significantly increased after restoring blood flow to the ischemic region via reperfusion, and for many years, the edema occurring after MI in the post-ischemic region has been believed to remain stable for at least one week [10,11]. However, post-I/R myocardial edema has recently been shown to follow a consistent bimodal pattern in a pig model during the first week after infarction [12], which was later confirmed in MI patients [13]. In these studies, an initial edema was detected in the first 3 h after the reperfusion onset that dissipated at 24 h and was followed by a delayed wave showing 4–7 days after infarction [12,13]. Using mass spectrometry (MS)-based quantitative proteomics, we reported for the first time the early proteome alterations caused by I/R in the ischemic and remote myocardium [14]. The first edema wave was shown to involve the coordinated change of hundreds of proteins related to the activation of inflammation, wound healing, ROS generation, and the downregulation of cell junctions and glycolytic metabolism. The second edema wave was associated with the replacement of necrotic myocytes with collagen, extracellular matrix proteins, and fibroblasts [14]. 

Recent advances based on so-called “open” database search strategies have paved the way for the comprehensive and unbiased analysis of protein post-translational modifications (PTMs) with MS-based proteomics [15,16,17]. We have recently made improvements to this technique that allow not only the identification of the modified sites but also automated quantitative analysis based on a validated statistical model [18,19]. We have also developed FASILOX [20,21], an improved method for the quantification of reversibly oxidized Cys residues that would otherwise remain undetected in the open search approach. These technical advances provide a unique framework for the proteome-wide, in-depth study of the molecular alterations that take place upon I/R. 

In this work, we have applied these techniques to build the first quantitative map of protein PTMs that take place in the ischemic heart during the first 24 h of reperfusion in the clinically relevant pig infarction model. Our study reveals that the reversible oxidative protein damage brought about by MI takes place approximately six hours after the reperfusion onset, while the corresponding irreversible oxidative damage spans two differentiated waves. In addition, a role for neutrophil infiltration is suggested in the second wave, which is caused by reperfusion. Of note, ischemic preconditioning lessened oxidative protein damage, inflammatory reactions, and contractile dysfunction caused by I/R. Finally, the above-described biphasic oxidative damage pattern and protein alterations were reproduced in an I/R mouse model, where neutrophil depletion resulted in the reduction in both oxidative protein damage and protein alterations.

## 2. Materials and Methods

### 2.1. Study Design and Animal Experiments

Forty castrated male Large White pigs weighing 30–40 kg were used for the pig I/R experiments. The study design, previously approved by the institutional animal research committee, is summarized in Figure S1a. Closed-chest, 40 min mid-left anterior descending (LAD) coronary artery occlusion was performed in 36 pigs. Twenty-eight of these animals were sacrificed after 20 min, 40 min, 80 min, 2 h, 6 h, 12 h, or 24 h of reperfusion (n = 4 per time group). Four additional animals were sacrificed 2 h after artery occlusion without reperfusion (2 h NOR, Figure S1b), and another four animals were subjected to ischemic preconditioning prior to I/R and sacrificed after 24 h of reperfusion (24 h PC) (Figure S1c). The remaining four pigs were sacrificed with no intervention other than baseline cardiac magnetic resonance (CMR) and served as controls. CMR scans were performed at every follow-up stage until sacrifice. Animals were immediately euthanized after the last follow-up CMR scan, and transmural myocardial tissue samples from the ischemic area were rapidly collected for proteomics evaluation. 

For mouse I/R experiments, we used wild-type 8–13-week-old C57BL/6 mice. The study design is summarized in Figure S1d. Mice were subjected to 45 min occlusion of the LAD coronary artery as previously described [22] and sacrificed after 10 min, 20 min, 40 min, 80 min, 2 h, 6 h, 12 h, 24 h, or 48 h of reperfusion (n = 3 per time group). Three additional mice were sacrificed with no intervention and served as controls. Whole heart tissue was collected for proteomics evaluation. Neutrophil depletion (Figure S1e) was performed in mice as previously described [23] via Ly6G injection 24 h and 48 h prior to I/R intervention (45 min ischemia followed by 24 h reperfusion), immediately after which the animals were sacrificed (24 h I/R Ly6G group, n = 3). A second group of mice were injected with IgG antibodies as a vehicle, subjected to I/R, and sacrificed (24 h I/R IgG group, n = 3). A third group of mice with no injection were also subjected to I/R and sacrificed (24 h I/R NT group, n = 6). The three corresponding mouse groups without I/R intervention were included: Sham Ly6G (n = 3, sacrificed after Ly6G injections), Sham IgG (n = 3, sacrificed after IgG injections), and Sham NT (n = 6, sacrificed without previous injection). Whole heart tissue was collected for proteomics analysis.

### 2.2. Myocardial Infarction in Pig and Mouse Models

The I/R protocol followed with pigs has been detailed elsewhere [12]. Anesthesia was induced by intramuscular injection of ketamine (20 mg/kg), xylazine (2 mg/kg), and midazolam (0.5 mg/kg) and maintained by continuous intravenous infusion of ketamine (2 mg/kg/h), xylazine (0.2 mg/kg/h), and midazolam (0.2 mg/kg/h). Animals were intubated and mechanically ventilated with oxygen (fraction of inspired O_2_: 28%). Central venous and arterial lines were inserted, and a single bolus of unfractionated heparin (300 IU/kg) was administered at the onset of instrumentation. The LAD coronary artery, immediately distal to the origin of the first diagonal branch, was occluded for 40 min with an angioplasty balloon introduced via the percutaneous femoral route using the Seldinger technique. Balloon location and maintenance of inflation were monitored angiographically. After balloon deflation, a coronary angiogram was recorded to confirm the patency of the coronary artery. A continuous infusion of amiodarone (300 mg/h) was maintained during the procedure in all pigs to prevent malignant ventricular arrhythmias. In cases of ventricular fibrillation, a biphasic defibrillator was used to deliver non-synchronized shocks. In the pre-conditioning group, the angioplasty balloon was placed immediately distal to the first diagonal branch and inflated 3 times for 5 min with low pressure (4 to 6 atmospheres) inflations. At the end of the pre-conditioning protocol, the balloon was inflated for 40 min and then deflated to allow reperfusion [24]. Short follow-up (2 h) non-reperfused MI was achieved by maintaining balloon inflation until sacrifice, as described in [25].

The I/R protocol applied to mice was performed as described in [26]. Male 8–12-week-old mice were subjected to 45 min occlusion of the LAD coronary artery followed by reperfusion. For the LAD procedure, mice were intra-peritoneally anesthetized with ketamine (60 mg/kg), xylazine (20 mg/kg), and atropine (9 mg/kg). Fully asleep animals were intubated, and the temperature was maintained throughout the experiment at 36.5 °C to prevent hypothermic cardioprotection. Thoracotomy was then performed, and the LAD was ligated with a nylon 8/0 monofilament suture for 45 min. The electrocardiogram was monitored to confirm total coronary artery occlusion (ST-segment elevation) throughout the 45 min ischemia. Neutrophils were depleted in C57BL/6 males via intravenous injections of 50 μg anti-mouse Ly6G 24 and 48 h before the myocardial I/R procedure [23].

### 2.3. CMR Protocol and Analysis

A baseline CMR scan was performed immediately before MI and subsequently repeated at the corresponding post-infarction follow-up time points until sacrifice. The images were acquired with a Philips 3-Tesla Achieva Tx whole-body scanner (Philips Healthcare, Best, The Netherlands) equipped with a 32-element phased-array cardiac coil. The imaging protocol included a standard segmented cine steady-state free-precession (SSFP) sequence to provide high-quality anatomical references and assessments of left ventricular (LV) mass, wall thickness, and LV ejection fraction (LVEF); a T2W-STIR sequence to assess the extent of edema; a T2-gradient-spin-echo mapping sequence to provide precise myocardial T2 relaxation time properties; and a T1-weighted inversion recovery turbo field echo sequence acquired 10 to 15 min after the administration of gadolinium contrast (late gadolinium enhancement to assess infarct size (IS) [24]. CMR images were analyzed using dedicated software (QMass MR version 7.6, Medis, Leiden, The Netherlands) by two observers experienced in CMR analysis and blinded to group allocation. Detailed information about imaging protocol and parameters and CMR analysis can be found in [24].

### 2.4. Tissue Sample Preparation for Proteomics Analysis 

The procedure is depicted in Figure S2a. Protein extracts from homogenized tissue were obtained using ceramic beads (MagNa Lyser apparatus, Roche, Germany) in extraction buffer (50 mM Tris-HCl, 1 mM EDTA, 1.5% SDS, 50 mM iodoacetamide (IAM), pH 8.5). The protein extracts were quantified using the RC DC protein assay (Bio-Rad, Hercules, CA, USA). We applied the filter-based FASILOX technology [20], which allows simultaneous quantitative analysis of Cys redox alterations and protein abundance changes. Briefly, the proteins that had been extracted in the presence of the alkylating agent IAM, which blocks free (reduced) Cys thiol groups, were loaded on a FASP filter (Expedeon, San Diego, CA, USA) used as a reaction chamber to (i) wash away detergents and other contaminants that would hinder tryptic digestion, (ii) reduce oxidized Cys with 50 mM dithiothreitol, (iii) alkylate the nascent Cys thiol groups with 50 mM methyl methanethiosulfonate, and (iv) perform trypsin digestion of proteins. This procedure enables later assessment of the original redox state of Cys-containing peptides by MS based on the specific mass shift observed for each alkylating agent in the corresponding precursor ions and fragments. Protein digestion was carried out overnight at 37 °C with sequencing-grade trypsin (Promega, Madison, WI, USA) at a 1:40 (*w*/*w*) trypsin/protein ratio in digestion buffer (8 M urea in 100 mM Tris-HCl pH 8.5), after which the resulting tryptic peptides were recovered via centrifugation. Trifluoroacetic acid (TFA) was added to a final concentration of 1%, and the peptides were desalted on C18 Oasis HLB extraction cartridges (Waters Corporation, Milford, MA, USA) and dried down. The peptide samples were taken up in 1 M triethylammonium bicarbonate, and their concentration was determined using a Direct Detect IR spectrometer (Millipore, Billerica, MA, USA). Equal amounts of the resulting peptides were isobarically labeled with tandem mass tags (TMT) 10-plex reagents (Thermo Scientific, San Jose, CA, USA) following the manufacturer’s instructions. The labeled peptide samples were mixed appropriately (Figure S2b) and dried down.

Aliquots of the dried labeled peptide samples were taken up in 300 µL of 0.1% TFA and separated into five fractions using the high-pH reversed-phase peptide fractionation kit (Thermo Scientific). The spin column was equilibrated with 300 µL of acetonitrile (ACN) twice, followed by 2 × 300 µL of 0.1% TFA. Then, the samples were loaded onto the columns and centrifuged at 3000× *g* for 2 min. The columns were then washed with 300 µL of water. The bound peptides were eluted into five fractions with 300 µL of freshly prepared elution solutions: (1) 12.5% ACN, 87.5% triethylamine; (2) 15% ACN, 85% triethylamine; (3) 17.5% ACN, 82.5% triethylamine; (4) 20% ACN, 80% triethylamine; and (5) 50% ACN, 50% triethylamine. The so-obtained fractions were dried and stored at −20 °C until MS analysis.

### 2.5. Liquid Chromatography Tandem Mass Spectrometry Analysis 

The labeled peptide samples were taken up in 0.1% formic acid (FA) and analyzed via nano-flow liquid chromatography coupled to tandem mass spectrometry (LC-MS/MS) using an Easy-nLC 1000 HPLC system (Thermo Scientific) coupled on-line to a Q Exactive HF hybrid quadrupole-orbitrap mass spectrometer (Thermo Scientific). C18-based reversed phase separation was used with a 2 cm trap column (Acclaim PepMap100, 75 μm internal diameter and 3 μm particle size, Thermo Scientific) and a 50 cm analytical column (Easy-Spray column PepMap RSLC C18, 75 μm internal diameter and 3 μm particle size, Thermo Scientific). Peptides were loaded in buffer A (0.1% FA (*v*/*v*)) and eluted with a 240 min linear gradient of buffer B (90% ACN, 0.1% FA (*v*/*v*)) at 200 nL/min flow. The MS spectra were acquired in the orbitrap analyzer within the 390–1500 m/z range and 70,000 resolution. Higher-energy collisional dissociation MS/MS spectra were acquired for the top twenty ions in each full MS scan in a data-dependent acquisition mode using 30 normalized collision energy and 45 s dynamic exclusion with 30,000 resolution in the orbitrap.

### 2.6. Peptide and Protein Identification 

The SEQUEST HT algorithm integrated into Proteome Discoverer 2.1 (Thermo Scientific) was used for peptide identification from LC-MS/MS data. The MS/MS scans were matched against a concatenated target-decoy database containing either both pig and human protein sequences (UniProtKB/Swiss-Prot 2017_06 Release) or mouse protein sequences (UniProtKB/Swiss-Prot 2017_06 Release). The following parameters were selected for database search: trypsin digestion with two maximum missed cleavage sites; 800 ppm and 0.02 Da precursor and fragment mass tolerance, respectively; fixed TMT modification at the N-terminus and Lys; variable oxidation at Met; and variable carbamidomethylation and methylthiolation at Cys. Peptide identification from MS/MS data was performed using the probability ratio method [27]. The false discovery rate (FDR) was calculated based on the refined method after 15 ppm precursor mass tolerance postfiltering [28,29]. A 1% FDR threshold was considered for peptide identification. Peptides were assigned to the best protein proposed by the Proteome Discoverer algorithm.

### 2.7. PTM Identification and Annotation 

Unbiased PTM analysis relied on open database searches with Comet-PTM, as previously described [18]. Briefly, the Thermo Scientific LC-MS/MS raw files were converted to the mzML format using ThermoRawFileParser version 1.1.9 [30]. The mzML MS data were open-searched against the aforementioned pig/human or mouse UniProt concatenated target-decoy databases using the following parameters: precursor ion mass tolerance of 500 Da, fragment ion mass tolerance of 0.02 Da, trypsin digestion without missed cleavages, fixed TMT labeling at the N-terminus and Lys, and fixed Cys carbamidomethylation. Comet-PTM first calculates the mass difference (ΔM) between the candidate peptide sequence and the precursor ion detected by MS; then, the ΔM is iteratively added to each amino acid in the peptide sequence, and the position that yields the best correlation score is selected as the correct match. The so-obtained peptide-spectrum matches (PSMs) and the corresponding ΔM values were further analyzed using the publicly available SHIFTS algorithm [18] version 0.3.1 available at https://github.com/CNIC-Proteomics/SHIFTS-4/releases/tag/v0.3.1 (accessed on 10 January 2024). In brief, SHIFTS comprises a set of software modules that (i) sequentially recalibrate experimental mass and ΔM values using a high-quality PSM subset (0.15 minimum corrected XCorr and 20 ppm tolerance); (ii) model ΔM values into a histogram grouped by ΔM bins; (iii) assign PSMs to ΔM peaks (defined by the frequency and slope of each bin); (iv) calculate PSM global, local (1-Da binning), and peak false discovery rate (FDR); (v) match ΔM values against a list of known mass shifts (most of which are taken from the Unimod database [31]); (vi) check whether ΔM values can be explained by peptide truncation; and (vii) check if ΔM values have been located in the right position in the PSM.

### 2.8. Quantification at the Peptide and Protein Levels

Quantitative information was extracted from the MS/MS spectra stored in the raw LC-MS/MS files obtained with TMT-labeled samples using an in-house MSQL library-based R script. The quantification of peptide and protein abundance was performed using the SanXoT package [32], a publicly available (https://github.com/CNIC-Proteomics/SanXoT, accessed on 10 January 2024) implementation of the WSPP model [33], and the Generic Integration Algorithm (GIA) [19] that was used to compute protein and peptide log2 fold changes for the reperfusion time-course samples with respect to the baseline samples. Protein log2 ratios were expressed in units of standard deviation according to their estimated variance (Zq values). The spectrum, peptide and protein variances, and protein values were initially determined using only non-modified peptides; in a second step, the modified peptides were included in the analysis, which was performed using the variances and protein values calculated previously. Quantitative abundance changes at the peptide level were determined by the corresponding standardized variable (Zp values), which estimated the log2 ratio deviation between peptides and the proteins they originate from. 

For the analysis of coordinated protein changes, we used the Systems Biology Triangle (SBT) algorithm [19], which estimates standardized functional category changes (Zc values). The proteins quantitated were functionally annotated using the DAVID repository [34], which comprised a number of functional databases, including Gene Ontology, KEGG, and Panther, among others.

### 2.9. H_2_O_2_ Measurement 

H_2_O_2_ production was measured with an Amplex Red H_2_O_2_ assay kit (Molecular Probes; Invitrogen, Waltham, MA, USA) according to the manufacturer’s instructions. In brief, left ventricular blocks (30–50 mg) were incubated with Amplex Red (100 µmol/L) and horseradish peroxidase (1 U/mL) for 30 min at 37 °C in Krebs–HEPES buffer protected from light. The supernatant was then transferred to a 96-well plate, and absorbance was measured at 560 nm. Background fluorescence, determined in a control reaction without a sample, was subtracted from each value. H_2_O_2_ release was calculated using H_2_O_2_ standards and expressed as micromoles per milligram of tissue.

### 2.10. Statistical Analysis

For the statistical enrichment analysis of PTMs, a hypergeometric *p*-value was calculated [35] using the total population of PTMs as a reference population. Oxidized protein enrichment was determined by calculating a hypergeometric *p*-value [35] for the enrichment using the total count of oxidized PSMs as a frequency value and the total PSM count for each protein as a reference population. The statistical differences in peptides, proteins, and functional categories affected by oxidative modifications across the different animal groups were evaluated by Kruskal–Wallis and Mann–Whitney tests using the GraphPad Prism 7.02 software. Protein–protein interaction network analysis was performed using String software (version 12.0, https://string-db.org/ (accessed on 29 December 2023)). Principal Components Analysis (PCA) was performed using the R ‘stats’ package [36], and ggplot2 was used for data visualization [37]. Hierarchical Clustering Analysis (HCA) was performed using R [36]. First, we computed the distance matrix for the modified peptides with the R ‘factoextra’ package [38] using the Pearson correlation coefficient as a distance measure, after which the correlation matrix was sorted using the hierarchical clustering algorithm in the R ‘corrplot’ package [39].

## 3. Results

### 3.1. Myocardial Infarction Produces Reversible Oxidative Protein Damage after Six Hours of Reperfusion

In a preliminary approach to studying PTM alterations produced by MI in the heart, we applied FASILOX [20] to analyze whether reversible Cys oxidative modifications could be detected in the same infarct samples obtained in our previous pig infarction study [14]. Since these samples had been obtained at 2 h, 24 h, 4 days, and 7 days of reperfusion time, this analysis could not account for the rapid reversible Cys oxidation produced by mitochondrial ROS that was detected at very short times (5 min) using a similar redox proteomics approach in perfused heart models [21,40]. Interestingly, reversible Cys oxidation was transiently incremented at 2 h, peaking at 24 h reperfusion and returning to initial values at 7 days (Figure S3 and Table S1). This oxidation burst took place between the first and second edema waves previously described [12,13] and between the coordinated protein alterations observed in the infarcted tissue after 2 h and 4–7 days of reperfusion time [14], suggesting the existence of intermediate oxidative molecular events. 

**Figure 1:**
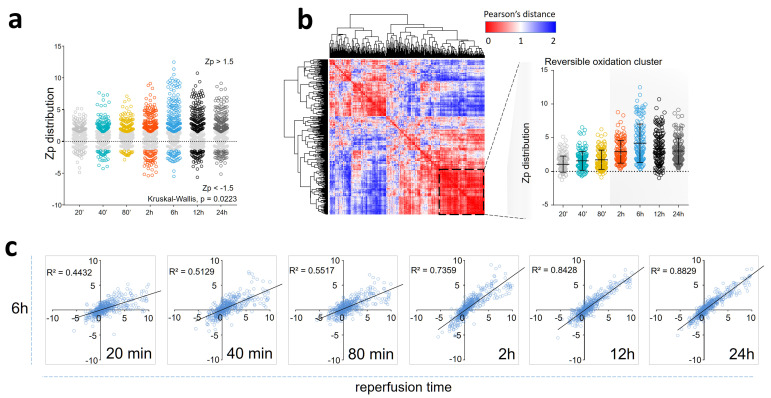
Reversible protein oxidation in the ischemic tissue during the first 24 h of reperfusion in the pig model. (**a**) Time course of reversible Cys oxidation changes, expressed in terms of Zp, the standardized log2 ratio at the peptide level, for the complete population of oxidized Cys peptides (n = 491). The *p*-values were calculated using the Kruskal–Wallis test. (**b**) Hierarchical Clustering Analysis (HCA) of reversible Cys oxidation (n = 491 peptides). The distance matrix was calculated using the Pearson correlation coefficient and sorted based on a hierarchical clustering algorithm. The inset displays the time course of reversible Cys oxidation for a cluster (highlighted by a dashed square) composed of 138 oxidized Cys peptides with increased abundance in the 2–24 h reperfusion time range with respect to baseline (Table S3). (**c**) Correlation analysis of oxidized Cys peptides at different reperfusion time points. The coefficient of determination (R^2^) for the correlation between Zp values (averaged from four biological replicates) at each time point and the corresponding values at 6 h of reperfusion time is shown.

To further explore the nature of these intermediate oxidative events, we designed a large-scale experiment including 32 animals and 7 intermediate time points using the pig I/R model (Figure S1a). Functional cardiac CMR results are displayed in Table S2. Redox proteomics analysis of the infarct samples (n = 4) allowed the quantification of 492 reversibly oxidized Cys peptides at each reperfusion time point, revealing a widespread increase in Cys oxidation within the first reperfusion day (Figure 1a). HCA disclosed a cluster of reversibly oxidized Cys peptides exhibiting similar time behavior (Figure 1b, Table S3), with increased abundance peaking at 6 h reperfusion time. In addition, correlation analysis showed that reversible Cys oxidation was remarkably reproducible across the different time points (Figure 1c). Our results support the existence of a reversible oxidation wave lying between the two edema waves previously detected via CMR (Table S2).

Protein enrichment analysis (Table S4) revealed that reversible oxidation affected mainly contractile, mitochondrial, and immune-system-related proteins (Figure S4), including the thin filament proteins ACTA2 and TNNC1; the two regulatory thick filament proteins MYL3, which is the regulatory light chain of myosin (MYH7), and MYBPC3; and ATP2A2, an enzyme involved in the regulation of the contraction/relaxation cycle. Oxidation also affected mitochondrial membrane carriers (mainly VDAC2), proteins implicated in the tricarboxylic acid cycle (like PDHA1, ACO2, and MDH2), and other regulatory and metabolism proteins. Proteins related to leukocyte-mediated immunity, including complement activation (mainly C3) and acute-phase response (AHSG and ITIH4), as well as circulating transporter proteins (TF), were also among the proteins most significantly affected by oxidation.

### 3.2. Myocardial Infarction Produces Two Different Waves of Irreversible Oxidative Protein Damage after Reperfusion

**Figure 2:**
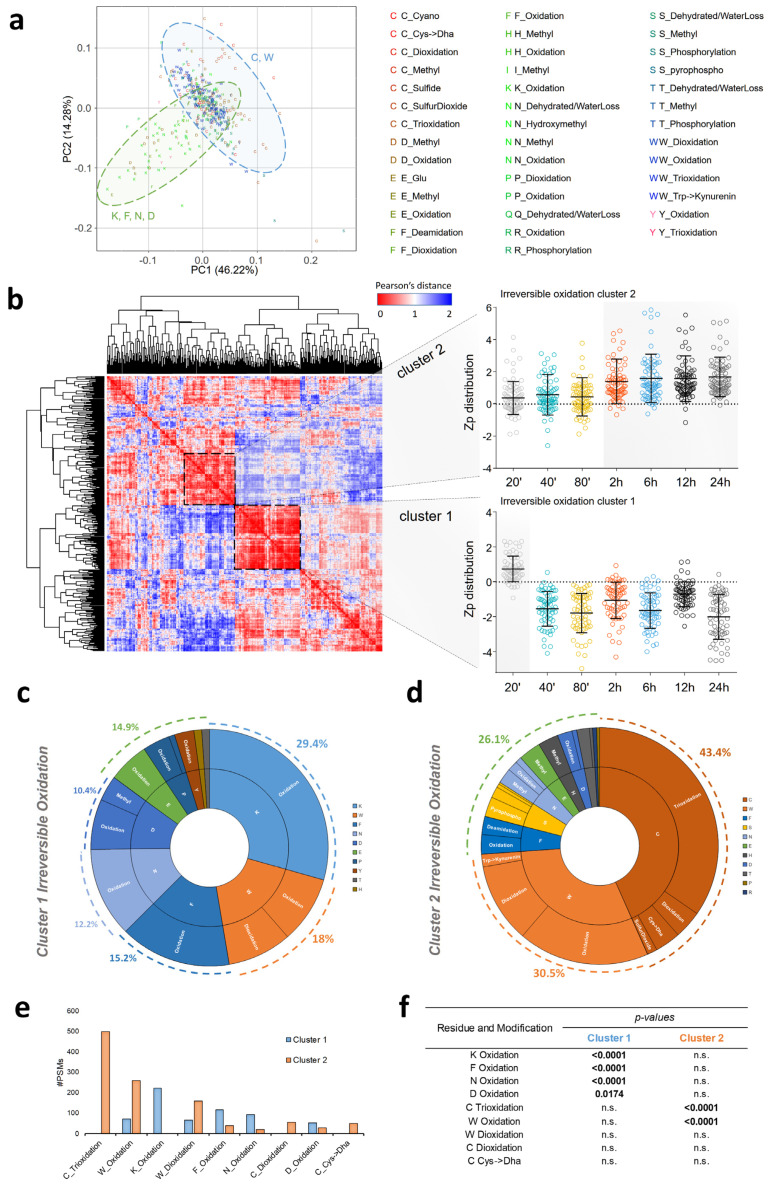
Irreversible protein oxidation events in the ischemic tissue during the first 24 h of reperfusion in the pig model. (**a**) Principal component analysis (PCA) of curated PTMs. A total of 409 peptides detected across the four biological replicates, with modifications located as expected according to Unimod, were included. The two shaded areas highlight two groups of PTMs: Lys, Phe, Asn, and Asp mono-oxidation (green: K, F, N, and D) and Cys and Trp mono-, di-, and trioxidation (blue: C and W). (**b**) HCA of curated PTMs (n = 409 peptides). The distance matrix was calculated using the Pearson correlation coefficient and sorted based on a hierarchical clustering algorithm. The insets display the time course of irreversible oxidation for two differentiated clusters (cluster 1 and cluster 2, highlighted by a dashed square) composed of irreversibly oxidized peptides. Zp values at each time point were averaged from four biological replicates with respect to baseline; to discard peptides with low correlation, only Zp series showing a maximum Zp > 0 in at least one time point were considered. The complete set of peptides composing clusters 1 and 2 is displayed in Tables S5 and S6, respectively. (**c**) PTM map of the early irreversible oxidation event that takes place 20 min after reperfusion (cluster 1, Table S5). The different PTM types and amino acid residues involved are indicated in the outer and inner borders, respectively. Border areas are proportional to the corresponding number of PSMs, and percentages were calculated with respect to the total number of PSMs composing cluster 1. (**d**) PTM map of the later irreversible oxidation event found in the 2–24 h reperfusion time range (cluster 2, Table S6). (**e**) PTM frequency (expressed as the number of PSMs) in clusters 1 and 2. Only modifications with more than 50 PSMs are shown. (**f**) PTM enrichment analysis. *p*-values are calculated as the probability of having more occurrences (PSMs) by chance alone in each cluster in comparison to the total population of PTMs and modified sites analyzed by HCA using the hypergeometric distribution. Only *p*-values from modifications with more than 50 PSMs are shown.

To elucidate the complete pattern of protein modifications triggered by the I/R process, the whole proteomics dataset was analyzed using Comet-PTM [18] and quantified by an integrated statistical model using the SanXoT package [32]. The open search approach yielded more than 11,500 modified peptide sequences where mass differences (ΔM) could be assigned to candidate PTMs. To obtain a curated list of modified peptides, we first discarded known chemical artifacts as well as ΔM values that were not found in the Unimod database [31]. We then selected a subset of peptides that were detected across the four biological replicates and bore modifications at expected amino acid residues according to Unimod. Finally, we also discarded peptides with ΔM values corresponding to known combinations of modifications to ensure that all measured peptide abundance changes are attributed to a single PTM. This restrictive filtering produced a curated list of 409 posttranslationally modified peptides coming from 149 proteins. PCA of the quantitative data revealed two separate populations of modified peptides (Figure 2a). Interestingly, the vast majority of these modifications were oxidative in nature; additionally, one of the populations contained peptides modified mostly at Cys and Trp, while the second population primarily consisted of peptides modified at Lys, Phe, Asn, and Asp. To further explore this behavior, we performed HCA to discern specific clusters of PTMs with similar profiles along the reperfusion time course. This analysis depicted two well-defined main clusters. The first cluster was composed of modified peptides that were increased at the shortest reperfusion time, i.e., 20 min (cluster 1 in Figure 2b; Table S5), and contained mainly irreversibly oxidized Lys, Trp, Phe, Asn, and Asp, which accounted for 85% of the cluster (Figure 2c). The second cluster was composed of modified peptides showing increased abundance in the 2–24 h reperfusion time range (cluster 2 in Figure 2b; Table S6), which overlapped in time with the reversible Cys oxidation peak. Most of the peptides that make up this cluster (74%) contained Trp and Cys modified mainly by mono-, di-, and tri-oxidation (Figure 2d). The relative proportion (in spectral counts) of the modified forms was completely different in the two peptide clusters (Figure 2e), and enrichment analysis demonstrated that cluster 1 was significantly enriched in peptides containing oxidized forms of Lys, Phe, Asn, and Asp, while cluster 2 was enriched in peptides with Cys trioxidation and Trp oxidation (Figure 2f). 

Enrichment analysis at the protein level (Table S4) showed that the early irreversible oxidation wave (cluster 1) affected mostly the principal thick-filament contractile protein myosin (MYH7), the cardiac muscle isoform of the regulatory light chain of myosin (MYL2), and MYOZ2, a sarcomeric binding protein involved in linking Z-line proteins in calcineurin signaling. In contrast, the second wave of irreversible oxidation (cluster 2) affected mainly the contractile regulator protein MYBPC3, the actin filament components ACTG1 and ACTN2, and the mitochondrial proteins CKMT2, MDH2, LDHB, and ALDOA (Figure S4). This wave also affected AHSG and ALB, among others. In general, the proteins affected by the second wave were similar in nature to those affected by the reversible oxidation wave, in agreement with their similar time patterns. In fact, several Cys residues affected by reversible oxidation were also detected as irreversibly oxidized (Figure S5). 

All these results show that the I/R process generates two separate oxidation waves with different molecular natures: an early wave showing immediately after the reperfusion onset, which gives rise to irreversible protein oxidation, and a late wave peaking several hours later, but before the second edema wave, which triggers both reversible and irreversible protein oxidation. 

### 3.3. Protein Abundance Changes during the Second Wave of Oxidative Damage Support a Role for Neutrophil Infiltration 

**Figure 3:**
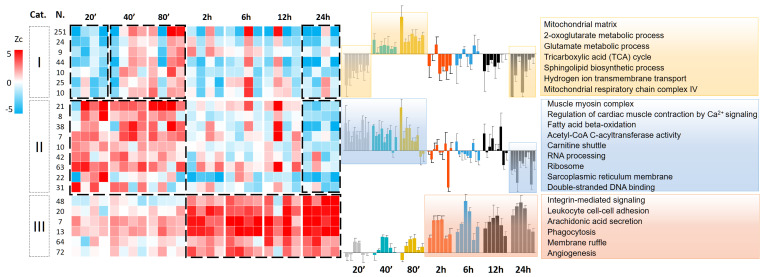
Systems biology analysis of early reperfusion in the ischemic myocardium in the pig model. Quantitative proteomics data were analyzed using the systems biology triangle (SBT) model to detect coordinated protein abundance changes along the I/R time course. The functional categories found to be significantly altered (FDR < 0.05) in at least one time point were classified into three groups. Group I includes mitochondrial metabolism categories found altered in the 20–80 min reperfusion time range and decreased 24 h post-I/R. Group II comprises biological processes increased early after the reperfusion onset (20–80 min), which are involved mainly in cardiac contraction and lipid metabolism proteins. Group III includes functional categories related to leukocyte migration, which increase in the 2–24 h reperfusion time range. Functional category abundance is expressed in terms of Zc, the standardized log2 ratio at the category level, calculated at each time point with respect to baseline. Table S8 displays the complete set of functional categories quantitated.

A systematic analysis of functional category alterations produced by coordinated changes in protein abundance was also performed using the SBT model [19]. This analysis showed a significant increase in proteins involved in processes and pathways related to leukocyte infiltration within the 2–24 h reperfusion time range (Figure 3), namely integrin-mediated signaling pathway components like ITGA1, ITGA5, and ITGB2; leukocyte cell–cell adhesion molecules like MSN and FERM3; arachidonic acid secretion proteins like ANNAX1; phagocytosis effectors like CD14, ELANE, and CORO1C; membrane ruffle components like GSN; and angiogenesis-related proteins like SERPINE1 and HRG. These results are in good agreement with the changes detected at similar time points in our previous proteomics study [14]. The upregulation of processes related to the immune response was coincident with the second oxidation wave, suggesting that this oxidative phenomenon could be caused by ROS released by neutrophils infiltrated in the ischemic lesion. In this regard, we found that the level of myeloperoxidase (MPO), the most abundantly expressed protein by neutrophils [41], was increased in the 6–24 h reperfusion time range, thereby correlating well with the second oxidation wave (Figure S6). 

Analysis of the samples obtained within the first two hours of reperfusion revealed early protein alterations that could not be detected in our previous proteomics study [14]. We observed a transient decrease in mitochondrial proteins (e.g., PDK4, DBT, IDH2, and DLST) at the earliest time point (20 min) that correlated with the first wave of irreversible oxidation events. We also observed a temporal increase in proteins related to the contractile machinery (e.g., MYH7, TTN, and CASQ2), energy production (e.g., ATP5A1, ATP5B, and CKMT2), and protein biosynthesis (e.g., RPS7, RPS9, and RPS13) within the 20–80 min reperfusion time range, probably reflecting a compensatory mechanism to recover heart activity. In addition, the ischemic myocardium showed a coordinated decrease in these mitochondrial and contractile-related proteins at a longer reperfusion time (24 h), in agreement with the results described in our previous work [14]. The complete set of quantitative results at the protein and functional category levels is presented in Tables S7 and S8, respectively.

### 3.4. The Second Wave of Oxidative Protein Damage Is Caused by Reperfusion

**Figure 4:**
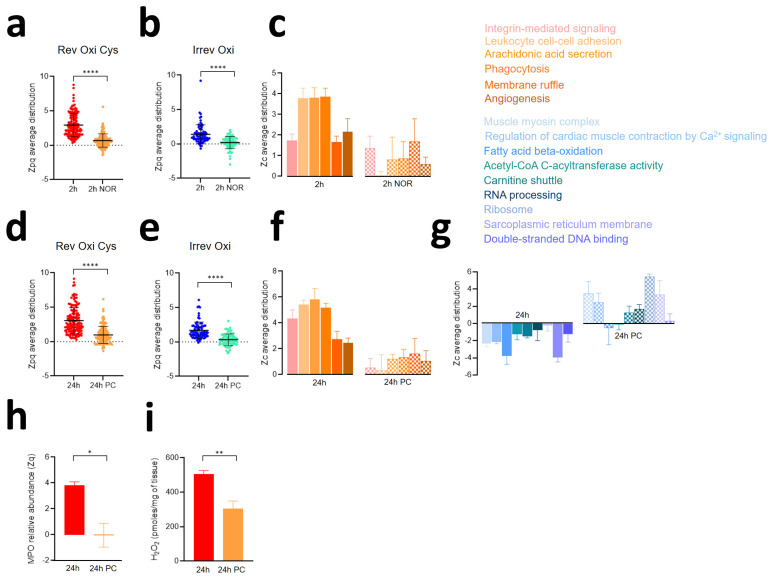
Effect of non-reperfusion and pre-conditioning on the processes associated with early reperfusion in the ischemic myocardium in the pig model. A group of 4 pigs was used for studying the effect of 2 h ischemia without reperfusion (2 h NOR; see Figure S1b). An additional 4-pig group was used to study the effect of 24 h I/R with ischemic preconditioning (24 h PC, see Figure S1c). The effect of 2 h NOR on reversible Cys oxidation (**a**) and irreversible oxidation (**b**) is shown, as is the effect of 24 h PC on reversible Cys oxidation (**d**) and irreversible oxidation (**e**). *p*-values were calculated using the Kruskal–Wallis test. Zp values at each time point were averaged from four biological replicates with respect to the baseline. The systems biology triangle (SBT) algorithm was employed to ascertain the effect of 2 h NOR (**c**) and 24 h PC (**f**,**g**) on coordinated protein abundance changes (FDR < 0.05). Zc values at each time point were averaged from four biological replicates with respect to the baseline. (**h**) MPO protein abundance in the 24 h PC group with respect to the 24 h I/R control group expressed in terms of Zq, the standardized log2 ratio at the protein level. For each condition, averaged Zq values from four biological replicates were calculated with respect to the baseline. (**i**) H_2_O_2_ level in the 24 h PC group compared to the 24 h I/R control group. *p*-values were calculated using the Mann–Whitney test. * *p* ≤ 0.05; ** *p* ≤ 0.01; **** *p* ≤ 0.0001.

Cardiac damage is produced by both the ischemia and the reperfusion phases, and the impact of reperfusion within the first few minutes has been extensively studied. To explore whether the second wave of protein oxidation is also related to reperfusion, we analyzed by proteomics a group of four pigs that had undergone 2 h of ischemia without later reperfusion (2 h NOR, Figure S1b). Functional cardiac CMR results (Table S2) indicated that the reperfused pigs had significantly higher myocardial water content and T2 relaxation times than the non-reperfused animals [25]. Both reversible Cys oxidation (Figure 4a) and the second wave of irreversible oxidation (Figure 4b) were significantly diminished in the non-reperfused animals, which showed oxidation values similar to those of the baseline group. In addition, the coordinated abundance changes of proteins related to leukocyte migration (integrin-mediated signaling pathway, leukocyte cell–cell adhesion, arachidonic acid secretion, phagocytosis, membrane ruffle, and angiogenesis) were markedly less increased in the non-reperfused group (Figure 4c). These results suggest that both the second oxidation wave and the stimulation of processes related to the immune response are direct consequences of reperfusion. 

### 3.5. Ischemic Preconditioning Reduces Oxidative Protein Damage, Inflammatory Reactions, and Contractile Dysfunction Caused by Ischemia-Reperfusion

To study whether the cardioprotective interventions aimed to reduce the impact of I/R affected the processes taking place during the second oxidative wave (2–24 h), we analyzed by proteomics a group of animals with ischemic preconditioning followed by 24 h reperfusion (Figure S1c) that showed reduced infarct size, edema size, T2, and increased LVEF as compared with non-preconditioned animals (Table S2) [24]. Preconditioning attenuated both reversible Cys oxidation (Figure 4d) and irreversible oxidation (Figure 4e) when compared to the non-preconditioned animals at 24 h of reperfusion. Consistently, preconditioning decreased the inflammatory processes related to leukocyte migration (Figure 4f). In addition, we found that the coordinated decrease in proteins related to mitochondria, sarcomere, energy production, and protein synthesis observed at 24 h was reversed in the preconditioned pigs (Figure 4g). MPO abundance (Figure 4h) and H_2_O_2_ levels (Figure 4i) were also diminished at 24 h in the preconditioned animals. H_2_O_2_ is the essential substrate necessary to convert MPO, which is abundantly expressed in neutrophils, into its reactive form. All these data suggest that preconditioning effectively prevents the molecular events occurring during the second oxidative wave, particularly those linked to neutrophil activation. 

### 3.6. The Biphasic Pattern of Oxidative Protein Damage and Protein Alterations Are Reproduced in a Mouse Model of Ischemia-Reperfusion

**Figure 5:**
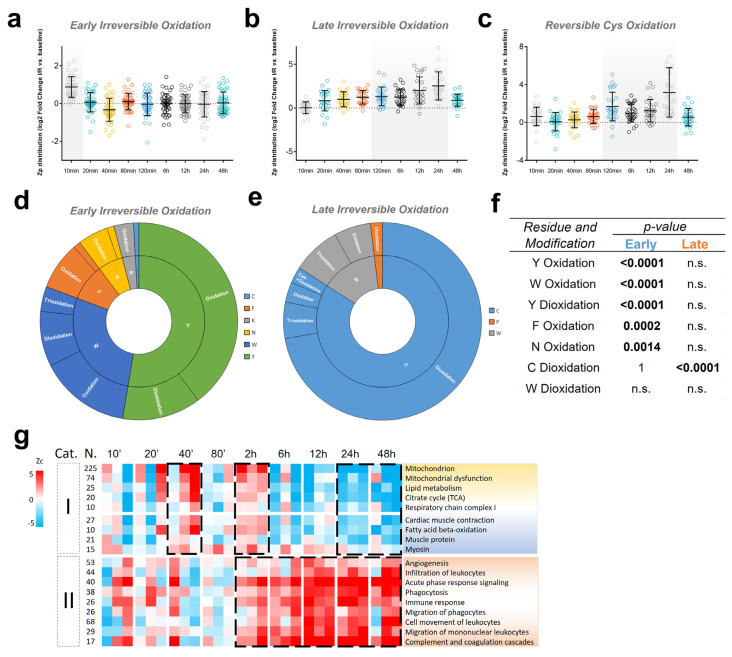
Oxidative events and functional protein alterations in the I/R mouse model. (**a**) Time course of irreversible oxidation showing an early wave at 10 min reperfusion time. Zp values at each time point were averaged from three biological replicates with respect to baseline; to discard peptides with low correlation, only Zp series showing a Zp average > 0.5 in at least one time point were considered (the complete set of modified peptides is displayed in Table S9). (**b**) Time course of irreversible oxidation showing a late wave in the 2–24 h reperfusion time range (the complete set of modified peptides is displayed in Table S10). (**c**) Time course of reversible Cys oxidation showing a wave in the 2–24 h reperfusion time range (the complete set of modified peptides is displayed in Table S11). (**d**) PTM map of the early irreversible oxidation event that takes place after 10 min of reperfusion. The different PTM types and amino acid residues involved are indicated in the outer and inner borders, respectively. Border areas are proportional to the corresponding number of PSMs. (**e**) PTM map of the late irreversible oxidation event that takes place in the 2–24 h reperfusion time range. (**f**) Irreversible oxidation enrichment analysis; *p*-values are calculated as in Figure 2f. (**g**) Quantitative proteomics data were analyzed using the systems biology triangle (SBT) algorithm to detect coordinated protein abundance changes along the I/R time course. The functional categories found to be significantly altered (FDR < 0.05) in at least one time point were classified into two groups. Group I includes mitochondrial and muscle contraction proteins, whilst group II components are mostly related to inflammation, immune system activation, and neutrophil infiltration. Functional category abundance is expressed in terms of Zc, the standardized log2 ratio at the category level, calculated at each time point with respect to baseline. n.s., not significant.

In anticipation of further validation interventions, we investigated whether the molecular changes taking place during I/R could be replicated in a mouse model of LAD coronary artery occlusion. For that, we conducted a time-course I/R experiment in mice, collecting infarct samples within the 10 min to 48 h reperfusion time window (Figure S1d) that were subjected to the same quantitative proteomics approach used for the pig I/R model. High-throughput PTM analysis of these mouse tissue samples confirmed the initial increase in irreversible oxidation at early time points (10 min) (Figure 5a and Table S9). These oxidative modifications were detected in Trp, Phe, Asn, and Lys, mirroring the residues affected in cluster 1 of the pig model (see Figure 2). Additionally, Tyr, which was the most affected residue in this model, also exhibited these modifications (Figure 5d,f). 

We detected a subsequent surge in protein oxidation, peaking between 2 and 24 h of reperfusion time, leading to irreversible modifications predominantly in Cys and Trp (Figure 5b,e,f and Table S10). Once more, these alterations closely mirrored the changes observed in cluster 2 of the pig model. The wave of reversible Cys oxidation (Figure 5c and Table S11) was also clearly detected in this model at similar times, which is also consistent with the data obtained in the pig model. Furthermore, upon examining the modified sites in the proteins primarily affected by oxidation, we found several homologous peptide sequences that underwent similar abundance changes over the time course and were affected by both irreversible oxidation (Figure S7a,b) and reversible Cys oxidation (Figure S7c) at identical sites in both the pig and mouse I/R models. 

Enrichment analysis at the protein level (Table S12) revealed that the early and late oxidation waves affected different proteins, as was observed in the pig model. On the other hand, we found a close similarity in the types of proteins affected by each wave between the two models; notably, Myh6, a thick filament protein that exhibits greater abundance than Myh7 in adult mice [42], and Ckm were prominently affected during the early oxidative wave in the mouse model. Likewise, sarcomeric proteins like Acta2, Myl3, and Myh8, together with mitochondrial and metabolism-related proteins such as Etfa, Slc25a4, Pdhb, Uqcrc1, and Ndufv1, were found to be impacted during the late oxidative wave. 

To further analyze the molecular alterations produced by I/R in the mouse model, we performed a systems biology analysis of the changes produced by the coordinated action of proteins along the reperfusion time course. We observed a significant increase in functional categories associated with leukocyte infiltration and immune response within the 2–48 h reperfusion time range (Figure 5g). These biological alterations correlated with an increase in MPO abundance (as measured by Zq, Figure S8) and also with the second oxidation wave (Figure 5b,c), consistent with observations in the pig model. This further suggests a role for neutrophil infiltration in these processes. We also observed in the mouse model a transient increase in categories related to mitochondrial functions and muscle contraction within the first two hours of reperfusion, followed by a subsequent decrease in these categories from 6 to 48 h (Figure 5g). Once more, these changes closely resembled those observed in the pig model (see Figure 3). Taken together, these results demonstrate that, despite the significant differences between the two animal models, they exhibit a notably reproducible pattern of molecular modifications over time following I/R.

### 3.7. Neutrophil Depletion Lessens Oxidative Protein Damage and Mitochondrial Protein Decrease Caused by Ischemia-Reperfusion in the Mouse Model

**Figure 6:**
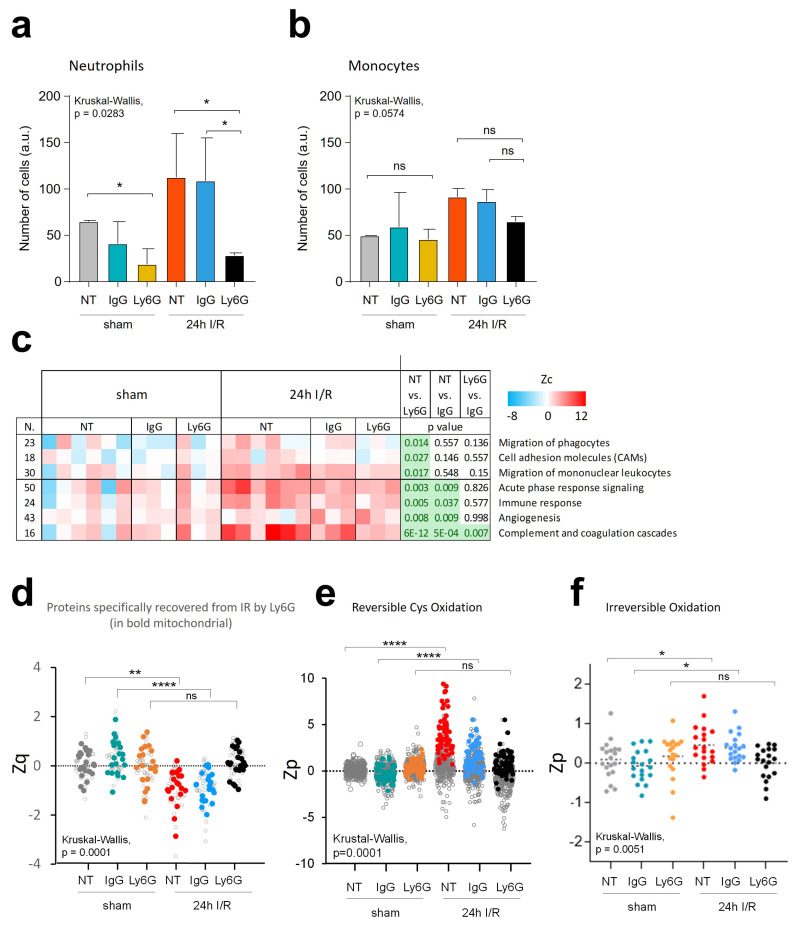
Effect of neutrophil depletion in the I/R mouse model after 24 h of reperfusion. Neutrophils were depleted using an anti-Ly6G antibody before I/R in three mice that were sacrificed after 24 h of reperfusion. Three mice used as negative controls were injected with IgG antibody as a vehicle and subjected to I/R (45 min ischemia followed by 24 h reperfusion). A third group of six mice with no injection (non-treated, NT) were also sacrificed after I/R. Twelve additional mice from the NT, IgG, and Ly6G groups were sacrificed without I/R intervention (sham groups). The experimental design is described in Figure S1e. Cell count of circulating neutrophils (**a**) and monocytes (**b**) in mouse peripheral blood. (**c**) Functional categories affected by neutrophil depletion in the I/R mouse model after 24 h of reperfusion. Functional category abundance values (Zc) are expressed as the log2 fold change of each condition with respect to the average of the NT-sham group. *p*-values were calculated using the Student’s *t*-test, and categories with an adjusted *p* < 0.05 (post hoc FDR Benjamini Hochberg) were considered significantly altered. (**d**) Relative abundance quantitation of selected proteins decreased after 24 h reperfusion in both the NT and IgG groups but remained at basal level in the Lys6G animals (open circles). Functional enrichment analysis showed a significantly increased abundance of mitochondrial proteins (filled circles). (**e**) Relative abundance quantitation of the total population of reversibly oxidized Cys peptides identified. Those peptides showing increased abundance 24 h after reperfusion in both the NT and the IgG groups but remaining at basal level in the Ly6G animals are highlighted with filled circles. The complete set of reversibly oxidized Cys peptides can be found in Table S13. (**f**) Relative abundance quantitation of irreversibly oxidized peptides in infarcted and sham-operated mice. The complete set of modified peptides is displayed in Table S14. The abundance of oxidized peptides is expressed in terms of Zp, the standardized log2 ratio at the peptide level, which was calculated in each condition (NT, IgG, and Ly6G) with respect to a pool of the sham-operated NT group (n = 6). *p*-values were calculated using the Kruskal–Wallis test. * *p* ≤ 0.05; ** *p* ≤ 0.01; **** *p* ≤ 0.0001; ns, not significant.

To elucidate the role of neutrophils in the molecular alterations observed upon I/R, we performed an I/R experiment in neutrophil-depleted mice. Neutrophil depletion was performed by anti-Ly6G injection, and IgG was used as a vehicle (Figure S1e). Depletion with anti-Lys6G resulted in a significant reduction in circulating neutrophil counts (Figure 6a), with negligible effect on blood monocytes (Figure 6b) when measured at 24 h reperfusion time. These findings agree with previously reported results [43]. The systems biology analysis of coordinated protein alterations showed a significant decrease in functional categories involved in the migration of leukocytes and phagocytes as well as cell adhesion molecules (CAMPs) in the heart samples from depleted mice but not in the IgG-treated controls, confirming that the anti-Lys6G antibody produced specific neutrophil depletion. Other functional categories related to generic immune response and inflammation were decreased in both the depleted (Ly6G) and control (IgG) animals with respect to the sham group (Figure 6c). This trend aligns with the anticipated immunomodulatory effects resulting from the injection of immunoglobulins, a phenomenon observed in various other animal models [44]. These were the only functional categories significantly affected by neutrophil depletion, according to our systems biology analysis, while the remaining categories sensitive to I/R (Figure 5g) were not altered. To further explore this point, we inspected the subpopulation of proteins that decreased at 24 h reperfusion in both non-treated and IgG-treated animals but maintained basal levels in the anti-Lys6G-treated animals (Figure 6d, open circles). These proteins specifically protected from I/R by neutrophil depletion were found to be significantly enriched (*p* < 0.0005) in mitochondrial proteins but not in other functional categories and comprised a large part of the subpopulation (Figure 6d, filled circles). These results indicate that while neutrophil depletion by the anti-Lys6G antibody did not confer complete protection, it was able to protect a significant proportion of mitochondrial proteins from the deleterious effects of I/R on this organelle.

Consistently, we observed that peptides exhibiting reversible oxidation at Cys that were increased at 24 h of reperfusion showed a diminished or non-significant increase in the anti-Lys6G-treated group. This effect was barely detected in the IgG-treated group (Figure 6e and Table S13). A similar trend was observed with the irreversibly oxidized peptides, where the I/R-induced increase was notably hindered by anti-Lys6G treatment, this effect being significantly different from that observed in the IgG-treated group (Figure 6f and Table S14).

All these data provide compelling evidence that neutrophil depletion mitigates the molecular impact of I/R observed specifically at 24 h in the mitochondrion, significantly preventing oxidative damage induced by I/R in the mouse model. Nonetheless, we cannot rule out the potential involvement of additional mechanisms contributing to the observed I/R damage.

## 4. Discussion

Preclinical animal models (reviewed in [45]) have significantly advanced our understanding of the molecular events underlying MI and have enabled the development of therapeutic strategies to lessen its devastating effects. While the generation of ROS represents one of the extensively studied events during I/R, there remains limited molecular evidence regarding early reperfusion-induced oxidation thus far [46,47,48,49,50,51,52,53]. Free radicals have been detected after I/R in perfused rabbit, rat, or guinea pig hearts using resonance spectroscopy or fluorescent and chemiluminescent indicators [46,47,54,55,56]. ROS produced by I/R in perfused rabbit hearts have also been demonstrated to alter sulfhydryl content, bityrosine and lipid peroxidation products, and oxidized glutathione levels [48,49]. Increased ROS, carbonyl content, and lipid peroxidation products have also been detected in open-chest mouse I/R models [50,51,52,53]. However, these works provide limited information on the nature and localization of the molecules affected. Additionally, not all the indicators seem to reflect the same molecular processes [47], and different reperfusion times were considered, ranging from 10 min or less [46,47,48,50,54,55,56] to 15 min [49], 30 min [51], 4 h [52], or 24 h [53]. 

Considerable effort has also been made to unveil the specific nature of reversible (i.e., reducible) Cys oxidative PTMs involved in the I/R process using redox proteomics approaches [21,40,57,58,59,60,61]. Thus, increased reversible Cys oxidation has been detected, mainly in mitochondrial proteins, in the Langendorff murine heart after 5–30 min of reperfusion [60,61,62], in agreement with the fact that mitochondria are a major source of ROS at this stage. We have reported an increase in specific reversibly oxidized Cys residues after 5 min reperfusion in Langendorff rat heart mitochondria [40]. Additionally, we have analyzed the protective effect of preconditioning on reducing Cys oxidation in isolated rat mitochondria [21,40]. Regarding PTMs other than reversible Cys oxidation, within the context of the I/R process, the analysis of irreversibly oxidized Cys sites has been confined to quantifying a small number of these sites in Langendorff rat hearts following a 15 min reperfusion [62]. Moreover, only a few PTMs involving residues other than Cys, such as phosphorylation, glycosylation, acetylation, and protein cleavage, have been studied within this context [63,64,65,66,67,68,69,70,71]. Many of these studies have relied on low-throughput approaches (e.g., Western blot or electrophoretic protein separation) for the analysis of Langendorff ischemic heart tissue from rodents subjected to short reperfusion times. Consequently, our knowledge about the molecular processes that take place during I/R is still very scarce; in particular, little is known about the precise PTMs involved, notably their time progression—a critical aspect as PTMs could serve as valuable surrogate markers of I/R injury. 

In this study, we employed unbiased, state-of-the-art proteomics approaches [18] to conduct a comprehensive, hypothesis-free analysis of PTMs occurring in the ischemic heart upon reperfusion. Diverging from previous works that primarily focused on short reperfusion times, our analysis encompassed a series of time points spanning the initial 24 h after reperfusion onset. In addition, we complemented the study with redox proteomics analysis using the FASILOX approach [20], enabling us to specifically identify reversible Cys oxidation patterns throughout the I/R process. 

**Figure 7:**
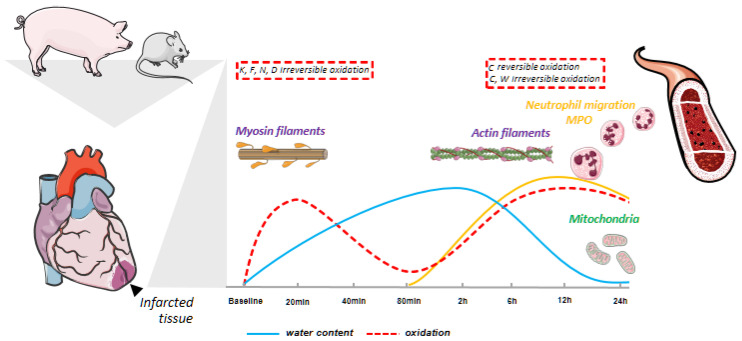
Schematic representation of the events that take place during the first reperfusion day in the ischemic pig myocardium. An early irreversible oxidation (red dotted line) wave is appreciated at the earliest reperfusion time (20 min) affecting Lys, Phe, Asn, and Asp residues from proteins essential for cardiac contraction; later, before the second edema wave (blue line), neutrophil infiltration to the cardiac lesion site (yellow line) triggered a second oxidation wave (red dotted line) peaking at 24 h reperfusion time that induced both irreversible oxidation at Cys and Trp residues and reversible Cys oxidation of mitochondrial, sarcomere, and inflammation-related proteins.

Our quantitative results revealed that the I/R process produces two well-differentiated oxidation waves: an early oxidation wave at the shortest reperfusion time considered (20 min) and a later oxidation wave within 6–12 h of reperfusion (Figure 7). Interestingly, the two oxidative events demonstrated markedly distinct molecular natures. The early oxidation wave induced irreversible oxidation of Lys, Phe, Asn, and Asp residues, primarily located within sarcomeric thick filament proteins. This phenomenon is likely a result of the release of ROS as a consequence of the opening of the MPTP. The late wave, peaking within 6–12 h of reperfusion time, involved reversible and irreversible oxidation at Cys and irreversible oxidation mainly at Trp residues. These modifications are predominantly located in proteins associated with contractile function, mitochondrial activity, and the immune system. 

In relation to the first wave, it is noteworthy that while we previously detected reversible Cys oxidation by redox proteomics in the mitochondrion as early as 5 min after reperfusion onset in both Langendorff rat hearts and isolated mitochondria [21,40], the present work, utilizing the same approach, did not detect reversible Cys oxidation at the earliest considered reperfusion time (20 min). This is due to the constraints of the in vivo pig model, which does not allow reperfusion times short enough to capture transiently oxidized Cys residues. However, our analysis successfully identified irreversibly oxidized Cys sites and revealed previously undocumented oxidations in other residues, particularly within mitochondrial and contractile proteins, which are the same kind of proteins that were affected in the reperfused heart models.

The late oxidative wave, observed within 6–12 h of reperfusion time, falls between the two edema waves described in the post-ischemic region, coinciding with increased infarct size [12,13,24]. This wave comprises both reversible and irreversible Cys oxidation, frequently occurring at identical sites. Of note, this is the first time that reversible Cys oxidation is detected at such long reperfusion times. The distinct types of proteins and specific sites impacted strongly suggest divergent origins of oxidative damage between the early and late oxidative waves. 

The results obtained with non-reperfused animals indicate that the late oxidation wave, which is characterized by decreased water content in the infarcted area and shorter T2 relaxation time [25], is caused by reperfusion. In addition, ischemic preconditioning followed by 24 h reperfusion resulted in a significant decrease in both reversible and irreversible Cys oxidation, together with the downregulation of inflammatory processes derived from leukocyte migration. Moreover, the preconditioning procedure reversed the coordinated decrease in proteins related to mitochondria, sarcomere, energy production, and protein synthesis and lessened MPO and H_2_O_2_ levels. This underscores that the cardioprotective effect of preconditioning, as evidenced by improvements in functional parameters such as LVEF, edema, and infarct size [24], also lessens the detrimental effects caused by the second oxidation wave, particularly those related to neutrophil activation. Taken together, all these results support the involvement of neutrophil infiltration in the late oxidative wave. While in this work we have focused on the effect of ischemic preconditioning and the absence of reperfusion, our results suggest that PTM analysis may become a useful tool to analyze the impact of other protective interventions at the molecular level. Such studies could result in novel approaches to rescuing reperfusion injuries. 

Heart size, heart rate, and vascular anatomy of large animals used to model MI (e.g., pig, mini-pig, sheep, and dog) are similar to those of humans, and this makes them the models of choice for translational medicine [72]. In addition, their larger heart size facilitates data collection (e.g., regional contractility and perfusion). On the other hand, advances in microsurgical and imaging/diagnostic procedures have made rodents the animal of choice for initial preclinical investigation [45]. Mice are favored due to the knowledge of their genome, the abundant variety of genetically modified strains, their short gestation, and their relatively low cost for breeding and maintenance; in contrast, large animal handling requires specialized facilities for housing and monitoring, as well as clinical standard surgery and imaging. To enhance the translation of findings to the clinical setting [73], this work utilized a closed-chest Large White pig I/R model to decipher the molecular hallmarks of I/R injury. Remarkably, the two-phase pattern of oxidative damage, together with the protein abundance and functional alterations, were clearly replicated in a C57BL/6 mouse model of surgically induced I/R despite the substantial physiological differences between the two animal species. This is of utmost interest not only for confirming our findings but also because it suggests that the molecular phenomena unveiled are conserved events in the mammalian cardiac cycle. Additionally, the mouse model allowed us to test the hypothesis of neutrophil infiltration in the second wave.

Results with the mouse model indicate that neutrophil depletion protects a significant fraction of mitochondrial proteins from I/R injury and hampers the increase in reversible Cys oxidation and irreversible oxidations induced by I/R. To our knowledge, this is the first proteome-wide depiction of the involvement of neutrophils in the oxidative damage associated with the I/R process. Thus, in the mouse I/R model, the level of MPO, which is abundantly expressed in neutrophils, peaks at 24 h of reperfusion; however, in the pig I/R model, the MPO level starts to increase at 6 h of reperfusion time, peaking at 12 h, and does not exactly overlap the maximum peak of neutrophil infiltration reported in the ischemic tissue [25]. Furthermore, in vitro assays with MPO and its oxidative by-products suggest that these molecules can quickly and selectively oxidize protein Cys and Trp residues [74,75,76,77], which aligns with our observations of these residues being affected in the second oxidative wave. Despite the fact that the level of MPO starts to increase at 6 h of reperfusion, whereas the second oxidation wave is increased at 2 h, our findings suggest that the MPO released by neutrophils recruited to the ischemic lesion might contribute to the emergence of the second oxidative burst. However, further research is necessary to determine which specific ROS are involved in the oxidative damage originated by this late oxidative wave. 

Some limitations of this study must be acknowledged. Firstly, while the pig is one of the most translatable animal models for the study of I/R injury because, unlike other mammals, its coronary artery anatomy and distribution resemble those of humans, extrapolation of results to patients is not straightforward. Thus, the time course of tissue changes in the reperfused myocardium is slightly shorter in pigs as compared to humans [78]. Moreover, with the pig I/R model, it is not possible to detect reversibly oxidized Cys residues at very short times after reperfusion; however, we have been able to detect irreversible Cys oxidations at the times used in our study. In addition, while pig ischemic myocardium samples were devoid of necrotic tissue, whole heart samples were used in the mouse I/R model. The use of homogenized tissue in both animal models for the proteomics experiments prevents the differentiation of protein and PTM abundance across different cell types or myocardial layers. Another limitation lies in the challenge of detecting certain PTMs (such as labile or hydrophobic modifications) using the shotgun LC-MS/MS approach employed. This approach also tends to exhibit a bias toward peptides of higher abundance (originating from highly abundant proteins). Finally, whilst Comet-PTM open-search pinpoints PTM sites with high accuracy (~85%), some modifications are expected to be incorrectly mapped; additionally, the method might fail with peptides bearing more than one PTM [18]. 

## Data Availability

The mass spectrometry proteomics data have been deposited to the ProteomeXchange Consortium via the PRIDE partner repository [79] with identifiers PXD045771 and PXD045759 for the time-course experiments with pig and mouse I/R models, respectively, and PXD045756 for the neutrophil depletion experiment with the mouse I/R model.

## Supplementary Materials

The following supporting information can be downloaded at https://www.mdpi.com/article/10.3390/antiox13010106/s1.
