# Supplementary material for: Oxidative Post-translational Protein Modifications upon Ischemia/Reperfusion Injury
Source: Antioxidants (Basel). 2024 Jan 16;13(1):106. doi: 10.3390/antiox13010106 (PMC10812827; doi:10.3390/antiox13010106)
Supplement: Supplementary file 1 [file antioxidants-13-00106-s001.zip › Supplementary Figures.pptx]

## Slide 1
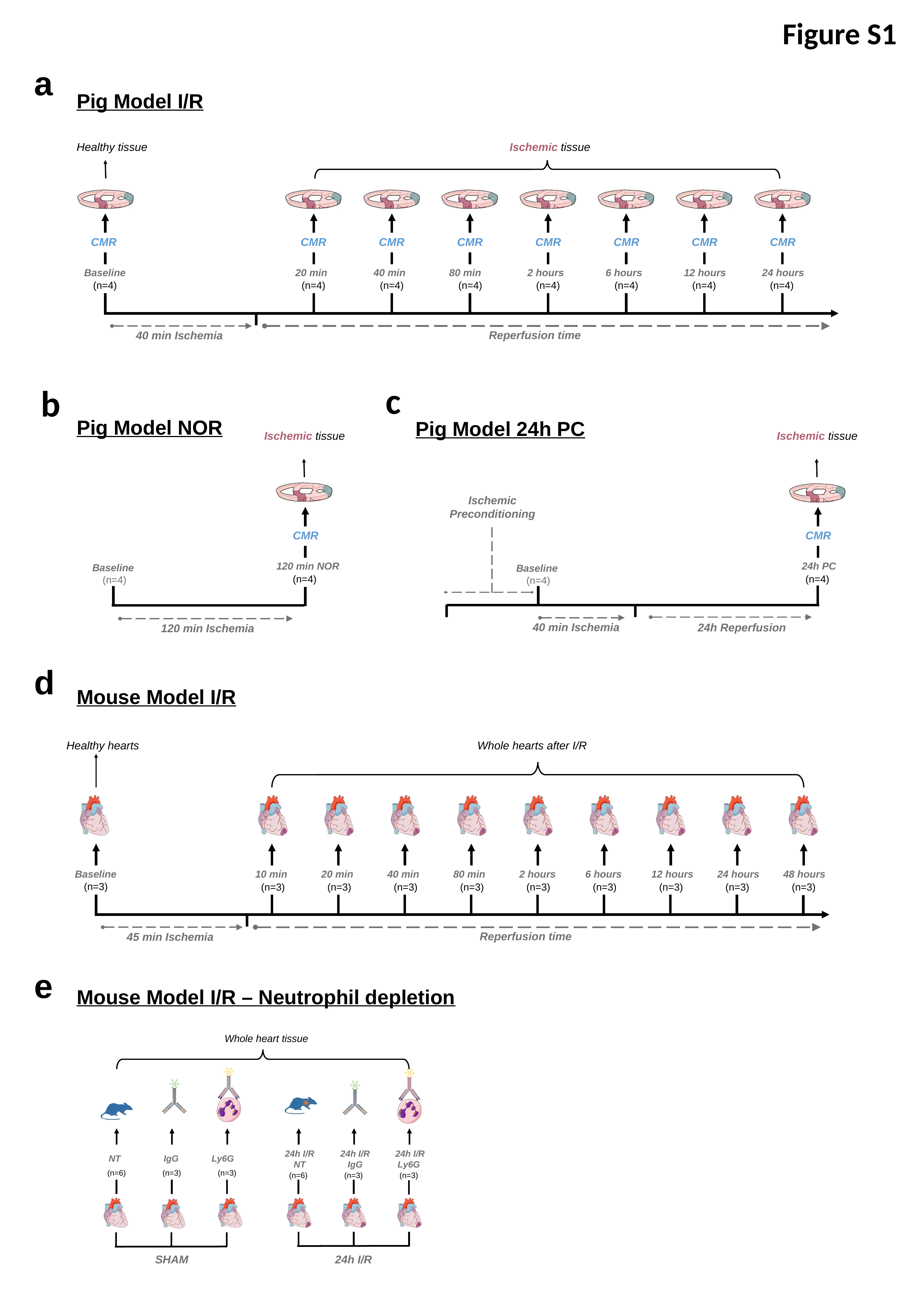

Figure S1
a
Pig Model I/R
Healthy tissue
Ischemic tissue
CMR
CMR
CMR
CMR
CMR
CMR
CMR
CMR
Baseline
20 min
40 min
80 min
2 hours
6 hours
12 hours
24 hours
(n=4)
(n=4)
(n=4)
(n=4)
(n=4)
(n=4)
(n=4)
(n=4)
Reperfusion time
40 min Ischemia
c
b
Pig Model NOR
Pig Model 24h PC
Ischemic tissue
Ischemic tissue
Ischemic Preconditioning
CMR
CMR
120 min NOR
24h PC
Baseline
(n=4)
Baseline
(n=4)
(n=4)
(n=4)
40 min Ischemia
24h Reperfusion
120 min Ischemia
d
Mouse Model I/R
Healthy hearts
Whole hearts after I/R
Baseline
10 min
20 min
40 min
80 min
2 hours
6 hours
12 hours
24 hours
48 hours
(n=3)
(n=3)
(n=3)
(n=3)
(n=3)
(n=3)
(n=3)
(n=3)
(n=3)
(n=3)
Reperfusion time
45 min Ischemia
e
Mouse Model I/R – Neutrophil depletion
Whole heart tissue
24h I/R NT
24h I/R IgG
 24h I/R Ly6G
NT
IgG
Ly6G
(n=6)
(n=3)
(n=3)
(n=6)
(n=3)
(n=3)
SHAM
24h I/R

## Slide 2
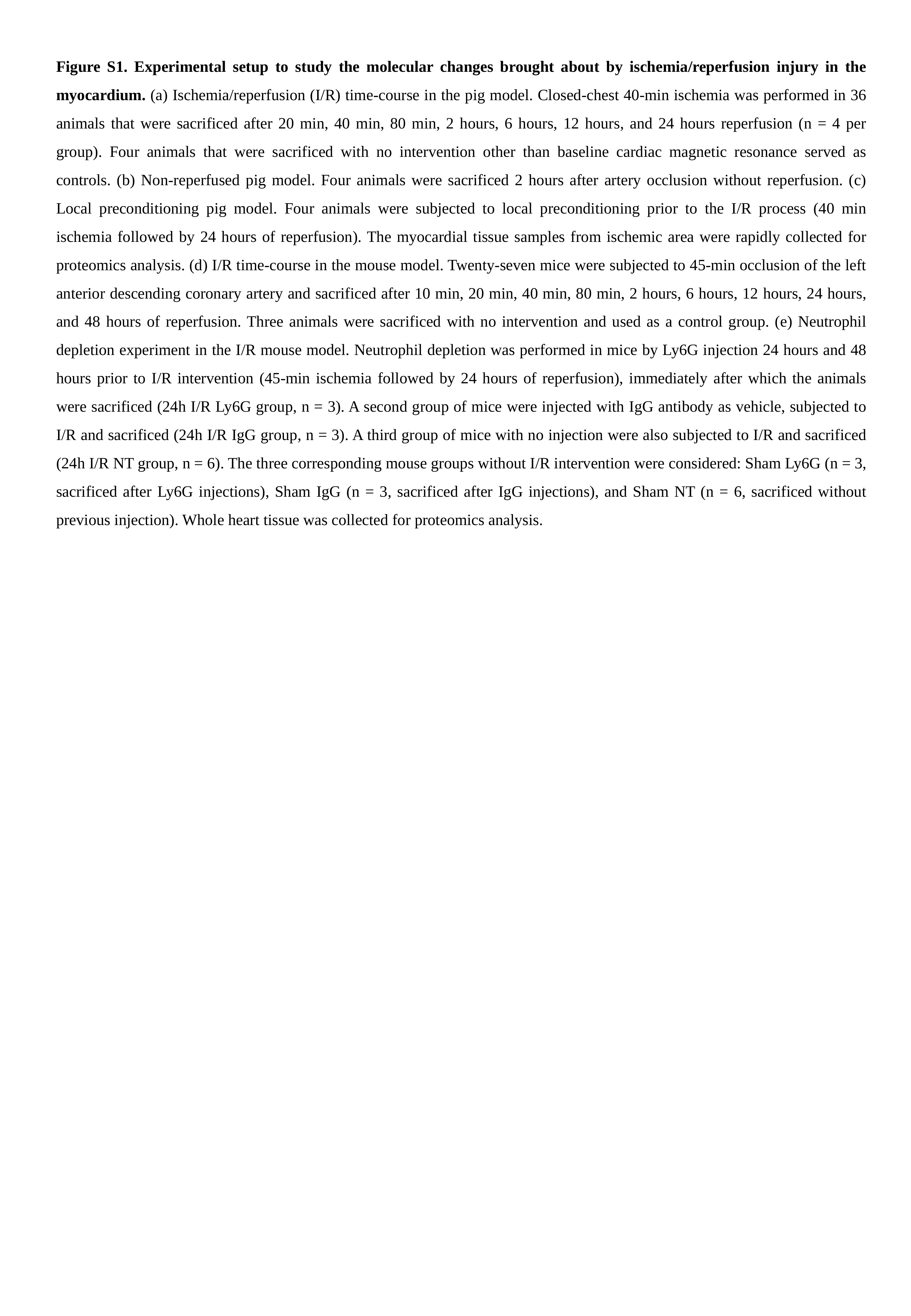

Figure S1. Experimental setup to study the molecular changes brought about by ischemia/reperfusion injury in the myocardium. (a) Ischemia/reperfusion (I/R) time-course in the pig model. Closed-chest 40-min ischemia was performed in 36 animals that were sacrificed after 20 min, 40 min, 80 min, 2 hours, 6 hours, 12 hours, and 24 hours reperfusion (n = 4 per group). Four animals that were sacrificed with no intervention other than baseline cardiac magnetic resonance served as controls. (b) Non-reperfused pig model. Four animals were sacrificed 2 hours after artery occlusion without reperfusion. (c) Local preconditioning pig model. Four animals were subjected to local preconditioning prior to the I/R process (40 min ischemia followed by 24 hours of reperfusion). The myocardial tissue samples from ischemic area were rapidly collected for proteomics analysis. (d) I/R time-course in the mouse model. Twenty-seven mice were subjected to 45-min occlusion of the left anterior descending coronary artery and sacrificed after 10 min, 20 min, 40 min, 80 min, 2 hours, 6 hours, 12 hours, 24 hours, and 48 hours of reperfusion. Three animals were sacrificed with no intervention and used as a control group. (e) Neutrophil depletion experiment in the I/R mouse model. Neutrophil depletion was performed in mice by Ly6G injection 24 hours and 48 hours prior to I/R intervention (45-min ischemia followed by 24 hours of reperfusion), immediately after which the animals were sacrificed (24h I/R Ly6G group, n = 3). A second group of mice were injected with IgG antibody as vehicle, subjected to I/R and sacrificed (24h I/R IgG group, n = 3). A third group of mice with no injection were also subjected to I/R and sacrificed (24h I/R NT group, n = 6). The three corresponding mouse groups without I/R intervention were considered: Sham Ly6G (n = 3, sacrificed after Ly6G injections), Sham IgG (n = 3, sacrificed after IgG injections), and Sham NT (n = 6, sacrificed without previous injection). Whole heart tissue was collected for proteomics analysis.

## Slide 3
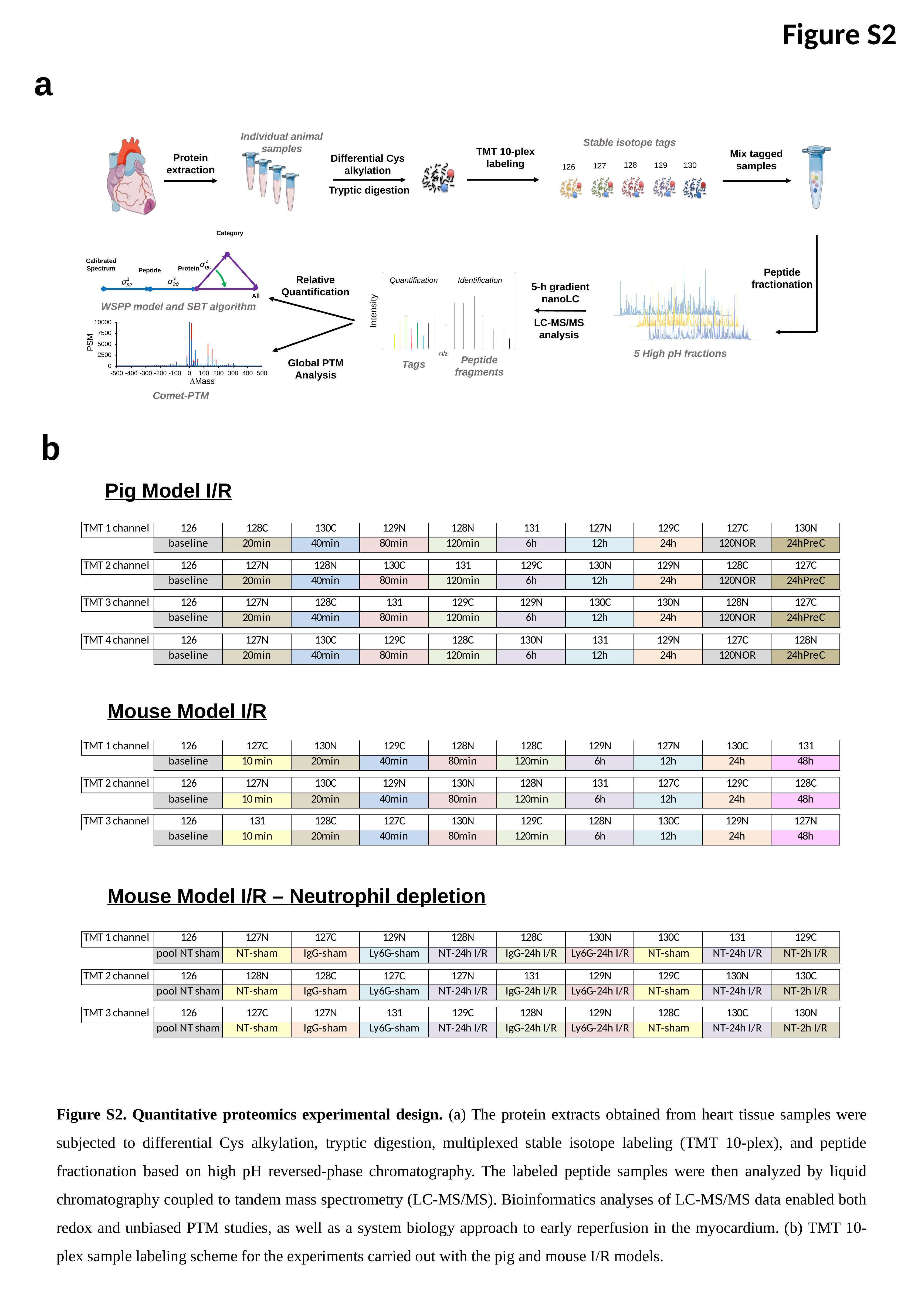

Figure S2
a
Individual animal
samples
Stable isotope tags
128
129
130
127
126
TMT 10-plex
labeling
Mix tagged samples
Protein extraction
Differential Cys
alkylation
Category
Calibrated
Spectrum
Protein
Peptide
All
Peptide fractionation
Relative Quantification
Intensity
Quantification
Identification
m/z
Peptide fragments
Tags
5-h gradient
nanoLC
WSPP model and SBT algorithm
LC-MS/MS analysis
5 High pH fractions
Global PTM Analysis
Comet-PTM
Tryptic digestion
b
Pig Model I/R
Mouse Model I/R
Mouse Model I/R – Neutrophil depletion
Figure S2. Quantitative proteomics experimental design. (a) The protein extracts obtained from heart tissue samples were subjected to differential Cys alkylation, tryptic digestion, multiplexed stable isotope labeling (TMT 10-plex), and peptide fractionation based on high pH reversed-phase chromatography. The labeled peptide samples were then analyzed by liquid chromatography coupled to tandem mass spectrometry (LC-MS/MS). Bioinformatics analyses of LC-MS/MS data enabled both redox and unbiased PTM studies, as well as a system biology approach to early reperfusion in the myocardium. (b) TMT 10-plex sample labeling scheme for the experiments carried out with the pig and mouse I/R models.

## Slide 4
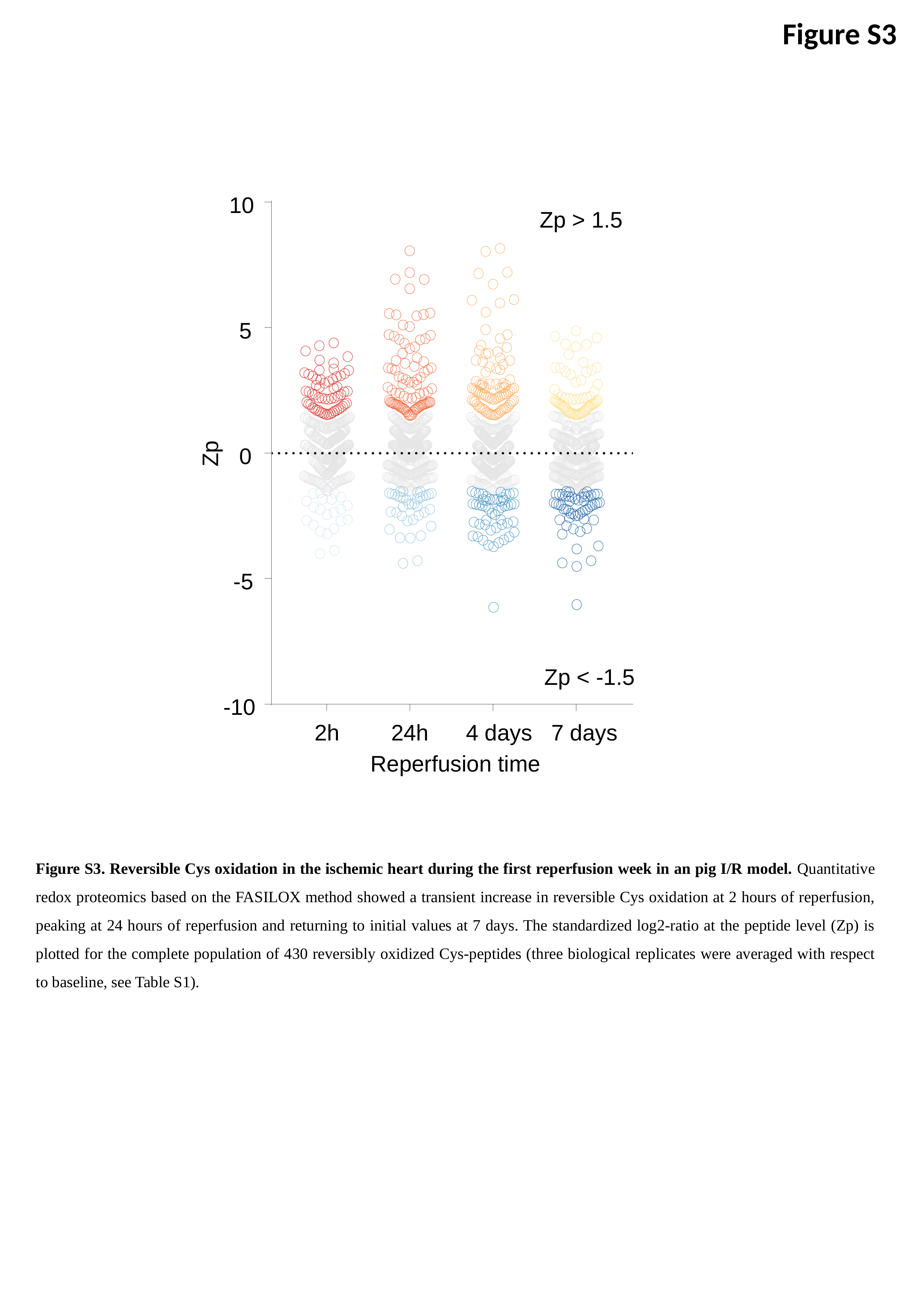

Figure S3
10
Zp > 1.5
5
0
-5
Zp < -1.5
-10
2h
24h
4 days
7 days
Reperfusion time
Zp
Figure S3. Reversible Cys oxidation in the ischemic heart during the first reperfusion week in an pig I/R model. Quantitative redox proteomics based on the FASILOX method showed a transient increase in reversible Cys oxidation at 2 hours of reperfusion, peaking at 24 hours of reperfusion and returning to initial values at 7 days. The standardized log2-ratio at the peptide level (Zp) is plotted for the complete population of 430 reversibly oxidized Cys-peptides (three biological replicates were averaged with respect to baseline, see Table S1).

## Slide 5
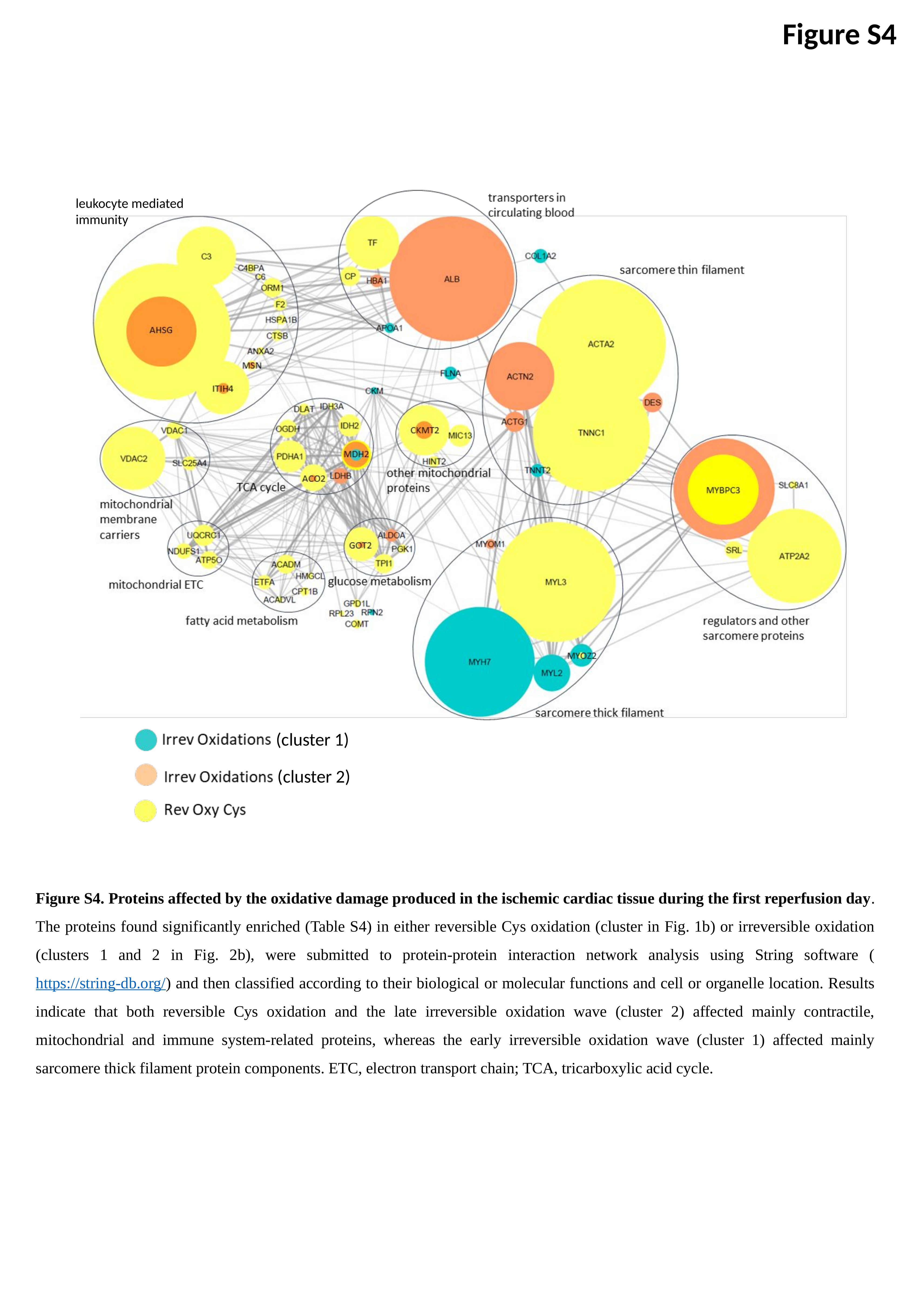

Figure S4
leukocyte mediated immunity
(cluster 1)
(cluster 2)
Figure S4. Proteins affected by the oxidative damage produced in the ischemic cardiac tissue during the first reperfusion day. The proteins found significantly enriched (Table S4) in either reversible Cys oxidation (cluster in Fig. 1b) or irreversible oxidation (clusters 1 and 2 in Fig. 2b), were submitted to protein-protein interaction network analysis using String software (https://string-db.org/) and then classified according to their biological or molecular functions and cell or organelle location. Results indicate that both reversible Cys oxidation and the late irreversible oxidation wave (cluster 2) affected mainly contractile, mitochondrial and immune system-related proteins, whereas the early irreversible oxidation wave (cluster 1) affected mainly sarcomere thick filament protein components. ETC, electron transport chain; TCA, tricarboxylic acid cycle.

## Slide 6
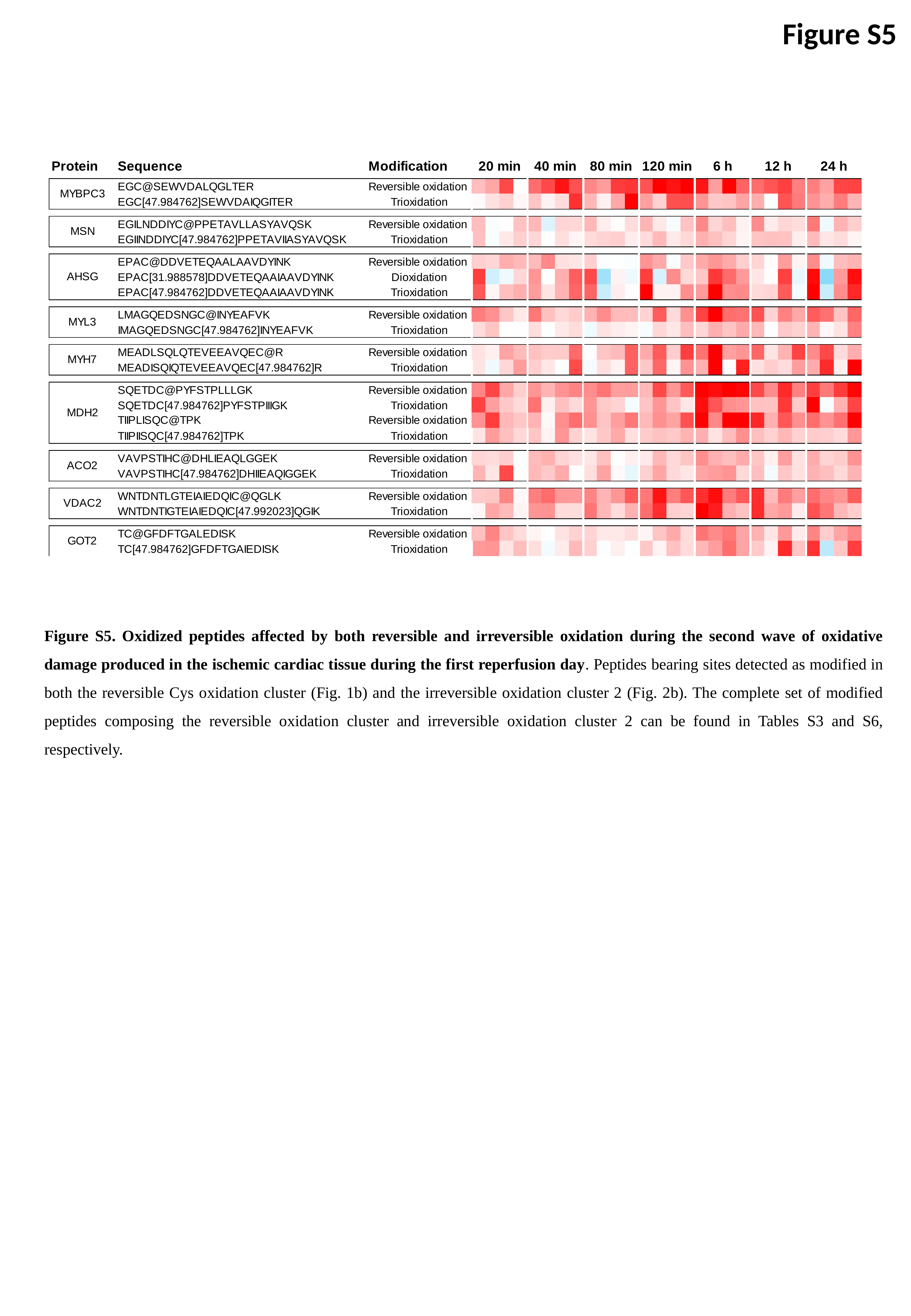

Figure S5
Figure S5. Oxidized peptides affected by both reversible and irreversible oxidation during the second wave of oxidative damage produced in the ischemic cardiac tissue during the first reperfusion day. Peptides bearing sites detected as modified in both the reversible Cys oxidation cluster (Fig. 1b) and the irreversible oxidation cluster 2 (Fig. 2b). The complete set of modified peptides composing the reversible oxidation cluster and irreversible oxidation cluster 2 can be found in Tables S3 and S6, respectively.

## Slide 7
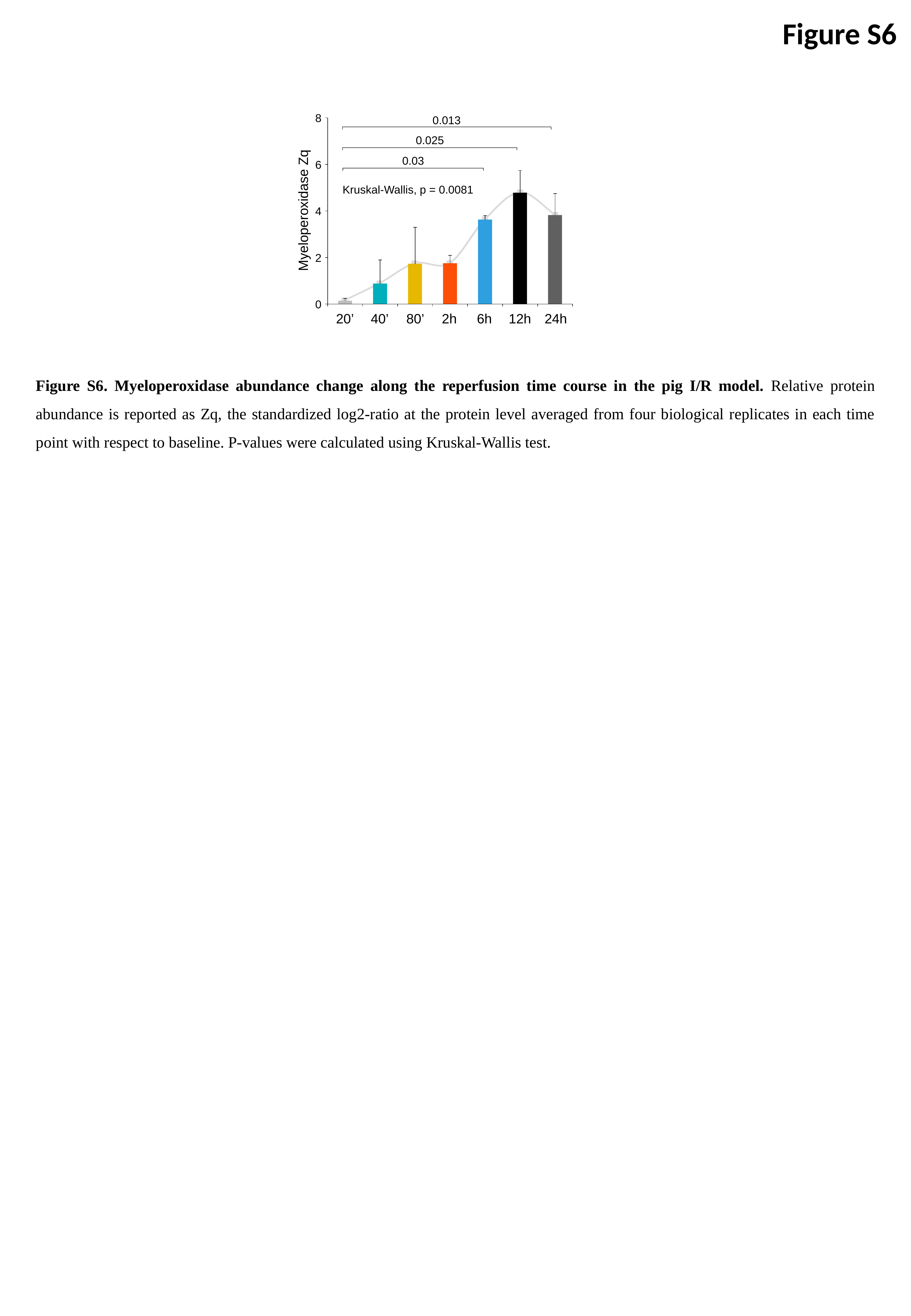

Figure S6
8
0.013
0.025
0.03
6
Kruskal-Wallis, p = 0.0081
Myeloperoxidase Zq
4
2
0
20’
40’
80’
2h
6h
12h
24h
Figure S6. Myeloperoxidase abundance change along the reperfusion time course in the pig I/R model. Relative protein abundance is reported as Zq, the standardized log2-ratio at the protein level averaged from four biological replicates in each time point with respect to baseline. P-values were calculated using Kruskal-Wallis test.

## Slide 8
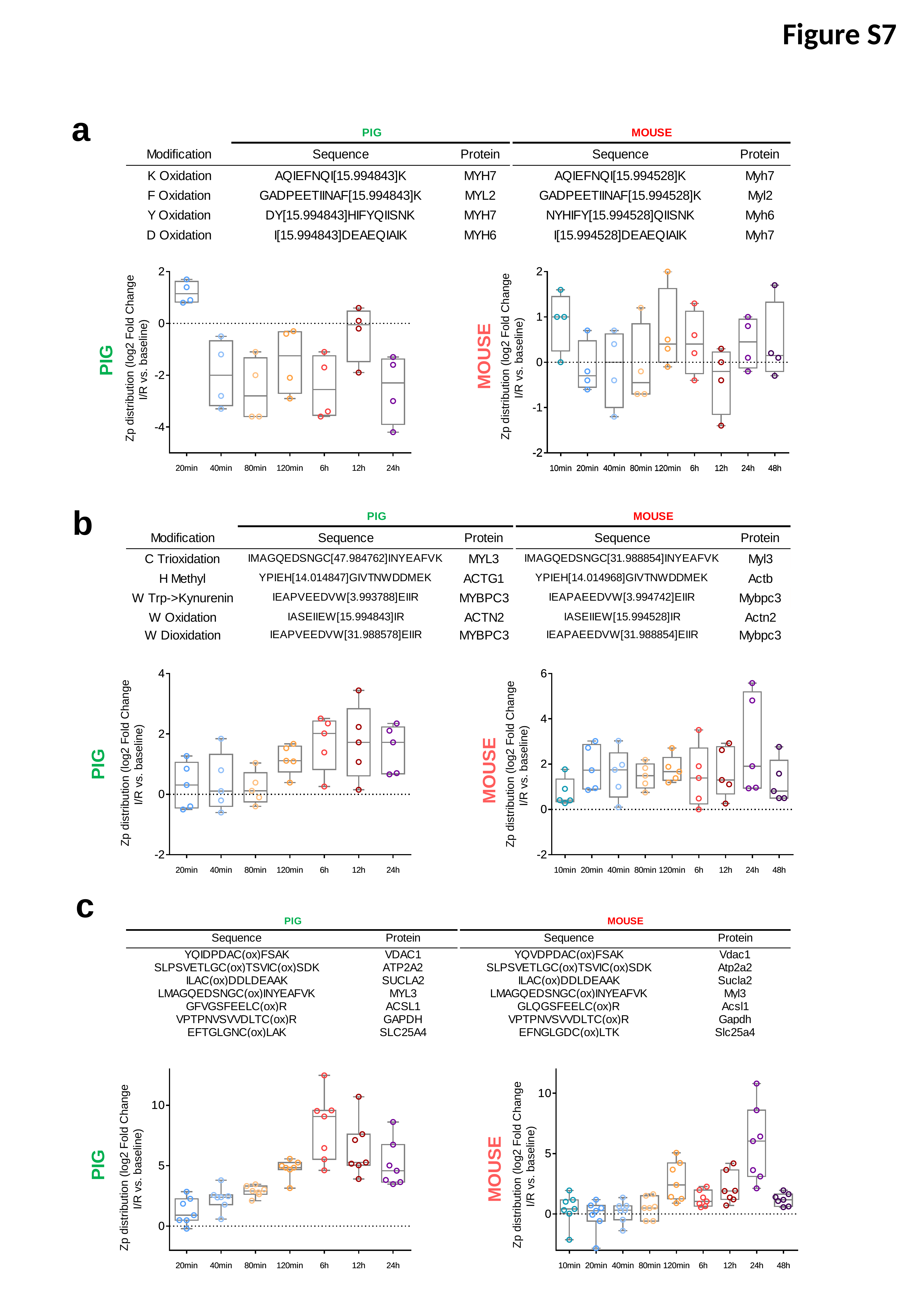

Figure S7
a
Zp distribution (log2 Fold Change I/R vs. baseline)
MOUSE
Zp distribution (log2 Fold Change I/R vs. baseline)
PIG
b
Zp distribution (log2 Fold Change I/R vs. baseline)
Zp distribution (log2 Fold Change I/R vs. baseline)
PIG
MOUSE
c
Zp distribution (log2 Fold Change I/R vs. baseline)
PIG
Zp distribution (log2 Fold Change I/R vs. baseline)
MOUSE

## Slide 9
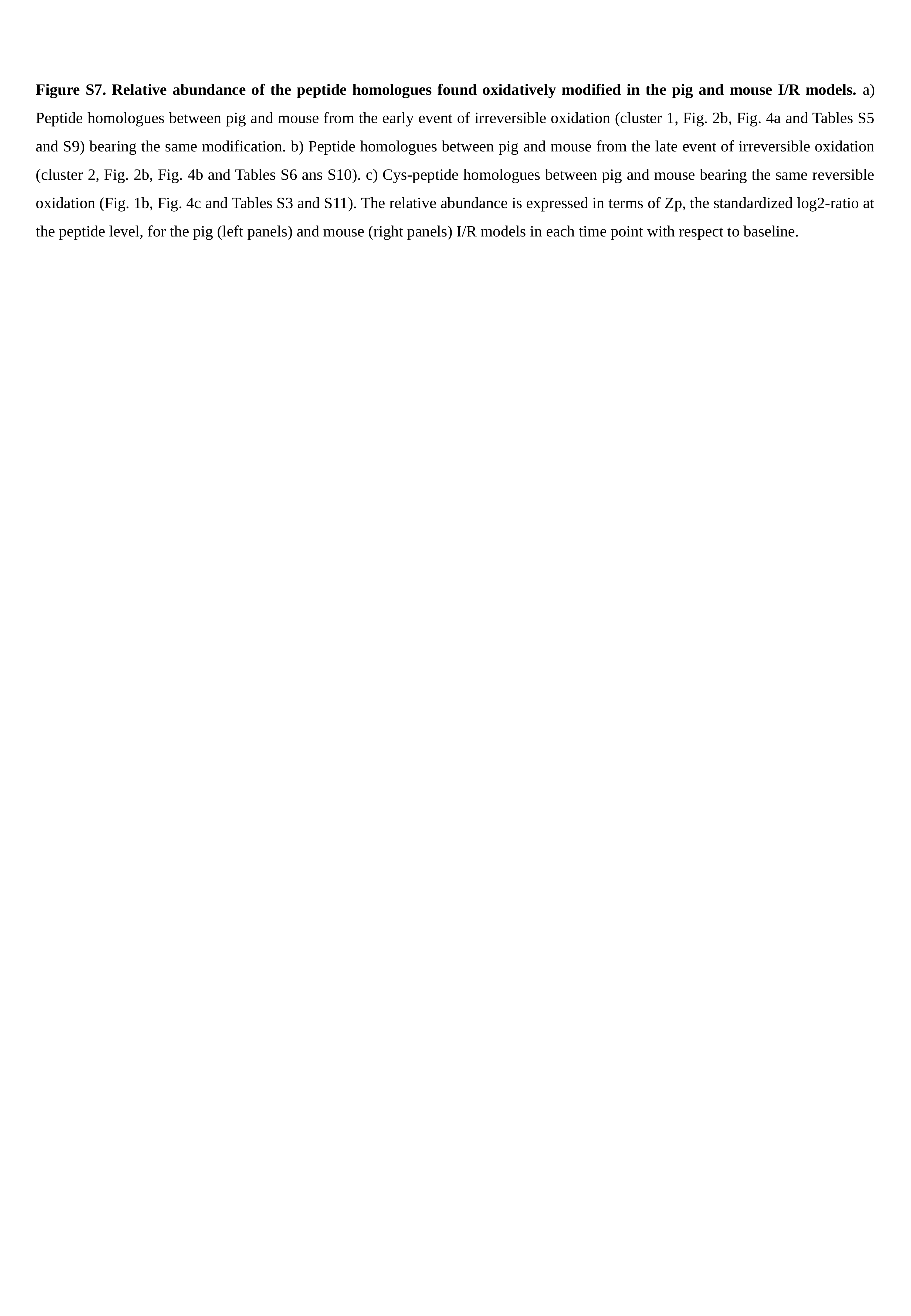

Figure S7. Relative abundance of the peptide homologues found oxidatively modified in the pig and mouse I/R models. a) Peptide homologues between pig and mouse from the early event of irreversible oxidation (cluster 1, Fig. 2b, Fig. 4a and Tables S5 and S9) bearing the same modification. b) Peptide homologues between pig and mouse from the late event of irreversible oxidation (cluster 2, Fig. 2b, Fig. 4b and Tables S6 ans S10). c) Cys-peptide homologues between pig and mouse bearing the same reversible oxidation (Fig. 1b, Fig. 4c and Tables S3 and S11). The relative abundance is expressed in terms of Zp, the standardized log2-ratio at the peptide level, for the pig (left panels) and mouse (right panels) I/R models in each time point with respect to baseline.

## Slide 10
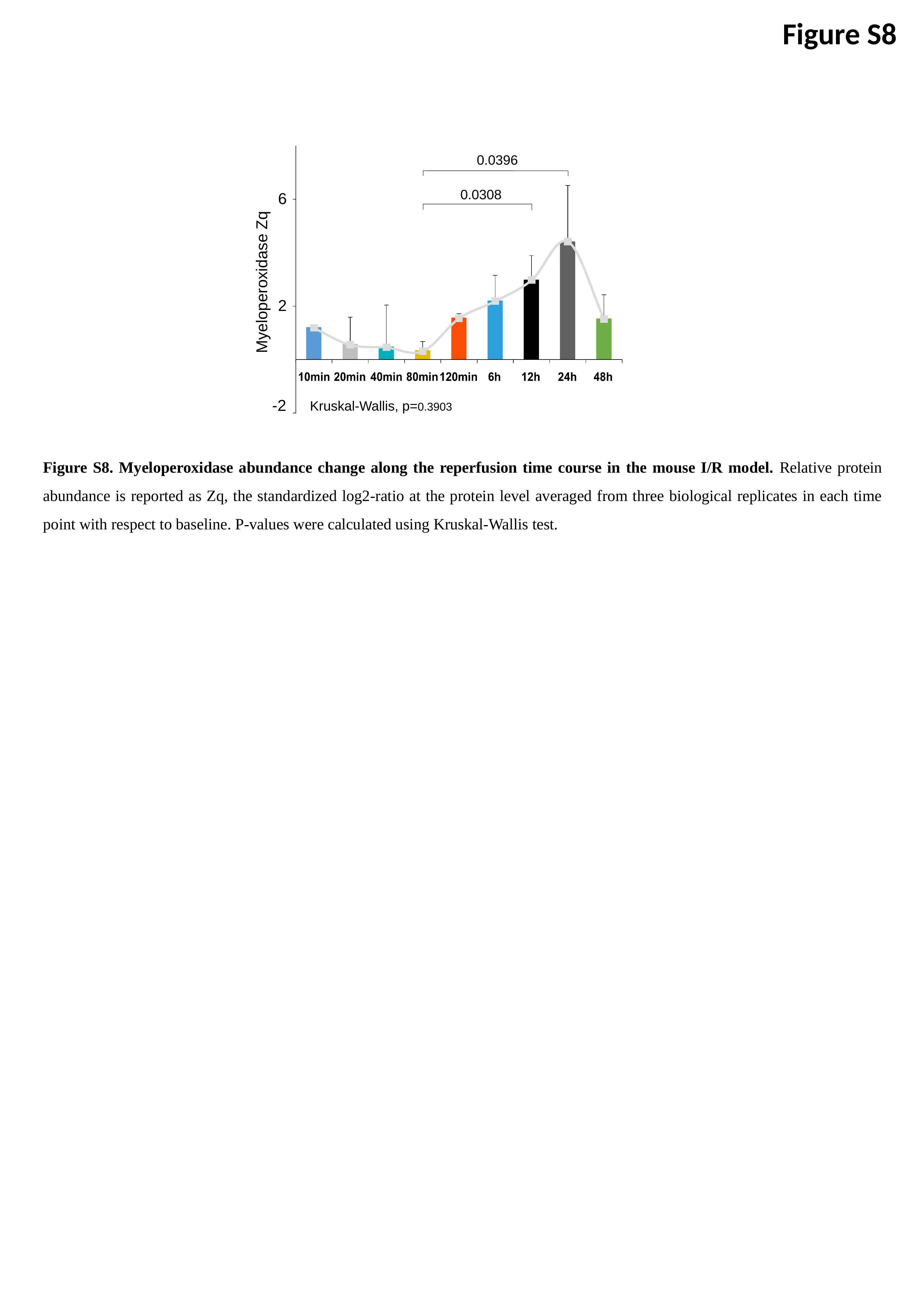

Figure S8
6
2
-2
0.0396
0.0308
Myeloperoxidase Zq
Kruskal-Wallis, p=0.3903
Figure S8. Myeloperoxidase abundance change along the reperfusion time course in the mouse I/R model. Relative protein abundance is reported as Zq, the standardized log2-ratio at the protein level averaged from three biological replicates in each time point with respect to baseline. P-values were calculated using Kruskal-Wallis test.
